# Isolation methods of exosomes derived from dental stem cells

**DOI:** 10.1038/s41368-025-00370-y

**Published:** 2025-06-16

**Authors:** Paras Ahmad, Nathan Estrin, Nima Farshidfar, Yufeng Zhang, Richard J. Miron

**Affiliations:** 1 Department of Research, Advanced PRF Education, Bradenton, FL USA; 2 Lake Erie College of Osteopathic Medicine School of Dental Medicine, Bradenton, FL USA; 3 Department of Periodontology, University of Bern, Bern, Switzerland; 4 Department of Oral Implantology, University of Wuhan, Wuhan, China

**Keywords:** Regeneration, Mesenchymal stem cells

## Abstract

Mesenchymal stem cells are highly regarded for their potential in tissue repair and regenerative medicine due to their multipotency and self-renewal abilities. Recently, mesenchymal stem cells have been redefined as “medical signaling cells,” with their primary biological effects mediated through exosome secretion. These exosomes, which contain lipids, proteins, RNA, and metabolites, are crucial in regulating various biological processes and enhancing regenerative therapies. Exosomes replicate the effects of their parent cells while offering benefits such as reduced side effects, low immunogenicity, excellent biocompatibility, and high drug-loading capacity. Dental stem cells, including those from apical papilla, gingiva, dental pulp, and other sources, are key contributors to exosome-mediated regenerative effects, such as tumor cell apoptosis, neuroprotection, angiogenesis, osteogenesis, and immune modulation. Despite their promise, clinical application of exosomes is limited by challenges in isolation techniques. Current methods face issues of complexity, inefficiency, and insufficient purity, hindering detailed analysis. Recent advancements, such as micro-electromechanical systems, alternating current electroosmosis, and serum-free three-dimensional cell cultures, have improved exosome isolation efficacy. This review synthesizes nearly 200 studies on dental stem cell-derived exosomes, highlighting their potential in treating a wide range of conditions, including periodontal diseases, cancer, neurodegenerative disorders, diabetes, and more. Optimized isolation methods offer a path forward for overcoming current limitations and advancing the clinical use of exosome-based therapies.

## Introduction

Extracellular vesicles (EVs) are nanoscale particles encased by a lipid bilayer that are released by nearly all cell types.^1,2^ EVs play a crucial role in intercellular communication by mediating the transfer of bioactive molecules, thereby influencing various biological processes such as immune modulation, tissue regeneration, and oncogenesis. These vesicles are classified into various subtypes based on their origin, dimensions, and molecular composition, with exosomes being one of the most extensively researched categories.^3^ Exosomes, generally measuring between 30 and 150 nm in diameter, arise from the endosomal pathway and are discharged into the extracellular space following the merger of multivesicular bodies with the plasma membrane.^4^ Their molecular contents – consisting of proteins, lipids, and nucleic acids such as microRNAs (miRNAs) and long non-coding RNAs (lncRNAs) – empower them to modulate cellular activities under both physiological and pathological conditions.^5^ The increasing focus on EVs, particularly exosomes, has highlighted their potential as valuable biomarkers and therapeutic agents with significant implications for regenerative medicine and tissue engineering.

The significance of exosomes in the field of regenerative medicine has gained increased interest due to their capacity to facilitate tissue regeneration and modulate immune reactions.^6^ Among the diverse origins of exosomes, mesenchymal stem cells (MSCs) have been thoroughly examined for their regenerative capabilities.^7^ Exosomes derived from MSCs (MSCs-Exos) have been demonstrated to enhance angiogenesis, regulate inflammatory responses, and promote tissue remodeling, rendering them promising candidates for therapeutic interventions.^8^ In contrast to direct MSCs transplantation, which is associated with challenges such as limited cell engraftment, potential immune rejection, and the risk of tumorigenicity, MSCs-Exos provide a cell-free therapeutic strategy characterized by lower immunogenicity and greater stability. The regenerative potential of MSCs-Exos is primarily attributed to their bioactive cargo, comprising cytokines, growth factors, microRNAs (miRNAs), and other signaling molecules that modulate cellular repair and tissue regeneration.

**Fig. 1:**
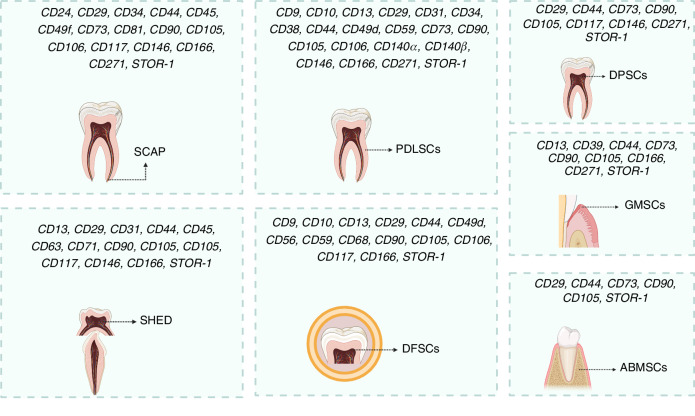
The surface markers and tissue origins of various dental stem cells, confirming their mesenchymal lineage and regenerative potential. While all share core mesenchymal stem cell markers (CD44, CD73, CD90, CD105, STRO-1), unique markers including CD24, CD140a/β, and CD31/CD63 define specialized roles in tissue repair, regeneration, and immunomodulation

Dental stem cells (DSCs), a distinct subpopulation of MSCs, have gained recognition as a promising source of exosomes for regenerative applications.^9,10^ Numerous subpopulations of DSCs have been explored to date, including stem cells from apical papilla (SCAPs),^11^ stem cells derived from human exfoliated deciduous teeth (SHEDs),^12^ dental follicle stem cells (DFSCs),^13^ gingival mesenchymal stem cells (GMSCs),^14^ periodontal ligament stem cells (PDLSCs),^15^ and dental pulp stem cells (DPSCs)^16^ (Fig. 1). Compared to MSCs derived from bone marrow or adipose tissue, DSCs can be obtained through minimally invasive procedures, thereby mitigating ethical concerns and minimizing donor-site morbidity.^17,18^ Notably, DSCs possess unique regenerative attributes, including a high proliferative potential, multipotency, and enhanced immunomodulatory properties.^19^ Beyond their ability to regenerate dental tissues, DSCs have also demonstrated the capacity to contribute to the repair and regeneration of various somatic tissues,^20–24^ highlighting their potential for broader therapeutic applications beyond the field of dentistry.

Although DSCs-Exos hold significant therapeutic promise, their clinical translation remains in its early stages, with several critical challenges that must be addressed to enable widespread adoption. Standardization of DSCs-Exos isolation and characterization protocols is essential to ensure consistency in yield, purity, and bioactivity.^8^ Additionally, scalable and reproducible bioprocessing techniques must be developed to facilitate cost-effective large-scale production for clinical applications.^25^ Optimizing delivery strategies, including the integration of biomaterial-based carriers, hydrogels, and scaffolds, is crucial for enhancing targeted exosome retention and therapeutic efficacy.^26^ Furthermore, rigorous evaluation of safety and regulatory considerations, including potential immunogenicity and off-target effects,^27^ is necessary through comprehensive preclinical and clinical studies to establish the reliability and safety of DSCs-Exos-based therapies.

## Comparative analysis of dscs-exos and mscs-exos

DSCs-Exos and MSCs-Exos share a common regenerative potential; however, distinct biological characteristics and therapeutic advantages set them apart. While both exosome types exhibit the ability to modulate immune responses, promote angiogenesis, and enhance tissue regeneration,^28,29^ DSCs-Exos have demonstrated unique advantages that warrant further investigation for their clinical applications. One of the key distinctions between DSCs-Exos and MSCs-Exos lies in their origin. Unlike MSCs-Exos derived from bone marrow or adipose tissue, DSCs-Exos are sourced from dental tissues through minimally invasive procedures, reducing ethical concerns and donor-site morbidity.^30,31^ Moreover, DSCs possess a higher proliferation rate and exhibit enhanced neurogenic and odontogenic differentiation capabilities,^32–34^ making their exosomes particularly suitable for applications in craniofacial and neural tissue regeneration.

Molecular profiling studies have revealed variations in the bioactive cargo of DSCs-Exos compared to MSCs-Exos. DSCs-Exos have been shown to contain a distinct repertoire of miRNAs, growth factors, and extracellular matrix proteins that contribute to their specialized regenerative effects.^28,35^ For example, DSCs-Exos are enriched in miRNAs associated with osteogenesis, neuroprotection, and angiogenesis, which may explain their superior potential in bone and nerve regeneration.^36^ In contrast, MSCs-Exos have been widely recognized for their immunomodulatory properties, making them valuable in autoimmune and inflammatory disease treatments.^37^ Furthermore, DSCs-Exos exhibit a favorable immunological profile, with studies suggesting a lower risk of eliciting immune rejection compared to MSCs-Exos. This immunoprivileged status enhances their feasibility for allogeneic transplantation without the need for extensive immunosuppression.^38^ Additionally, the extracellular matrix components and adhesion molecules present in DSCs-Exos may enhance their retention at injury sites,^30^ potentially improving therapeutic efficacy.

## Applications of exosomes in personalized medicine

For numerous reasons, exosomes have attracted considerable interest in scientific research as innovative vehicles for drug delivery. In contrast to synthetic nanoparticles, exosomes have demonstrated diminished toxicity and immunogenicity in preliminary investigations. These observations imply that exosomes may pose a lower risk of eliciting adverse reactions or immune responses when utilized as vehicles for drug delivery. Exosomes originate from cells and are constituted by lipid bilayers that closely resemble cellular membranes.^39^ This particular composition enhances the biocompatibility and stability of exosomes, enabling them to safeguard cargo molecules against degradation and unfavorable conditions within the body, such as enzymatic breakdown and acidic environments.^26^ Studies have indicated that exosomes can successfully reach their destination organs following circulation within the bloodstream. Furthermore, exosomes possess the capacity to deliver therapeutics through penetration of the blood-brain barrier.^40^ Consequently, extensive efforts have been undertaken to load a variety of therapeutic agents, thereby augmenting the therapeutic efficacy of these agents.^41^ A significant number of ongoing investigations are concentrated on optimizing the cargo loading of exosomes to fully exploit their potential as drug delivery systems. Diverse methodologies have been devised to incorporate therapeutic agents, including small-molecule pharmaceuticals and genetic materials, into exosomes.^39^

Two primary techniques employed to load drugs into exosomes are passive loading and active loading.^42^ The selection of technique may hinge upon the specific drug and its characteristics, alongside the intended release mechanism. Passive loading transpires when cells are cultured with the drug of interest, facilitating the natural incorporation of the drug into the exosomes during their formation. The cells internalize the drug, encapsulating it within exosomes, and subsequently release the drug-laden exosomes into the surrounding environment. This method is straightforward and facile to implement; however, it suffers from low loading efficiency and presents challenges in regulating the quantity of drug encapsulated within the exosomes. Passive loading is typically most effective for lipophilic drugs that can readily traverse cellular and vesicular membranes. Nevertheless, this approach may not be suitable for all drug categories. Alternatively, active loading entails the direct incorporation of the drug into isolated exosomes. This process is generally executed through various techniques that enhance the permeability of the exosomal membrane, such as exposure to detergents, electroporation, thermal shock, sonication, or the application of saponins. Active loading achieves superior loading efficiency relative to passive loading and permits more precise regulation of the drug loading quantity. However, this method can be intricate, and improper execution may compromise the process.^43^ In both approaches, the elimination of any unencapsulated drug following loading, utilizing diverse purification techniques such as ultrafiltration, size-exclusion chromatography (SEC), or ultracentrifugation, is essential.

Genetic engineering methodologies may also be employed to load genetic materials into exosomes. Cells can be genetically modified to express a protein or RNA of interest, which can subsequently be integrated into exosomes, during their formation.^42^ This approach can be employed for loading particular RNA or protein molecules into exosomes; however, necessitates intricate molecular biology strategies and might not be suitable for all kinds of therapeutic agents.^44^ Each of these approaches possesses benefits and limitations, and the best approach can rely on factors including the type of drug, the source of the exosome, and the intended drug delivery target.^45^

## Exosome isolation techniques

To enable the comprehensive investigation and utilization of these distinctive EVs, exosomes must be meticulously extracted from an array of interfering constituents and cellular debris.^46^ The methodologies utilized in the exosome isolation process should demonstrate exceptional efficacy and possess the capability to extract exosomes from various sample matrices. With the rapid progress in scientific research and technological advancements, numerous approaches have been developed for the isolation of exosomes in significant quantities and with high purity.^47^ Each method capitalizes on specific characteristics of exosomes, such as their size, morphology, density, and surface proteins to facilitate their isolation.^48^ Variants within each category also introduce distinct sets of benefits and limitations concerning exosome isolation (Table 1)^3,49–54^

**Table 1:** Comparison of most used isolation/extraction techniques of exosomes

Isolation method	Principle	Sample volume	Time/h	(Recovery/Yield)/%	Purity	Advantages	Limitations	References
Differential ultracentrifugation	Sedimentation rate	100 mL	> 4	5 to 20	Medium	Gold standard; suitable for large-volume specimens; Comparatively cheap	Might damage exosomes; low yield; cumbersome operation; time-consuming	^49,50^
Gradient ultracentrifugation	Shape, size, and density	≈ 1 mL	> 16	10 to 40	High	Avoiding exosomal damage; high purity	Cumbersome operation; preliminary preparation; labor-intensive	^49,50^
Ultrafiltration	Particles with varying molecular weight and size	> 100 μL	> 4	≈ 30	High	Easy; no requirement of special reagents and equipment	Loss of exosomes of small particle diameter; clogging on filtering membrane	^3,51^
Size-exclusion chromatography	Particles with varying molecular weight and size	≈ 1 mL	0.3	≈ 40 to 80	High	Simple; economical; maintain biological structure and function	Lipoprotein contamination; packing and special column are needed	^3,52^
Polymer-based precipitation	Sedimentation rate	≈ 100 μL	2 to 18	5 to 30	Low	Simple; suitable for large-volume samples	Potential contaminants	^53,54^
Immunoaffinity-based capture	Surface marker expression	> 100 μL	4 to 20	> 90	High	High specificity for exosome subtypes isolation	Extensive; depending on antibody’s specificity	^53,54^

The selection and modification of isolation approaches ought to be tailored according to the specific biological fluid from which exosomes are extracted. An effective isolation approach must concentrate the exosomal signal for analysis while avoiding contamination from other molecules, such as EVs, lipoproteins, and protein aggregates. For example, blood plasma encompasses high-density lipoprotein (7–15 nm), LDL (20 to 40 nm), very-low-density lipoprotein (30–90 nm), and chylomicrons (200–600 nm), all of which exhibit similarities to EVs regarding density and size. This complicates the exosomal isolation process, as conventional methods struggle to exclude lipoprotein contamination.^55^ In this regard, it has been observed that LDL may co-purify with exosomes isolated from both platelet concentrations and blood plasma.^56^ Hence, it is recommended to utilize SEC in conjunction with a density gradient to effectively segregate EVs from lipoproteins.^57^ Additionally, the presence of fetal bovine serum (FBS) EVs and contaminants in cell culture media poses challenges to exosomal isolation in vitro. The exosomes found in FBS have been documented to possess biological activity. Hence, their co-isolation could disrupt subsequent in vivo or in vitro investigations. Numerous methods have been implemented to address this issue, such as culturing cells in serum-free media, which might lead to significant cellular starvation, altering cellular behavior, composition, and the number of exosomes secreted. An alternative strategy involves utilizing commercially available exosome-depleted or home-generated FBS. Another possibility is the application of bile salts, synthetic agents, pituitary extracts, or platelet lysates.^58^

**Fig. 2:**
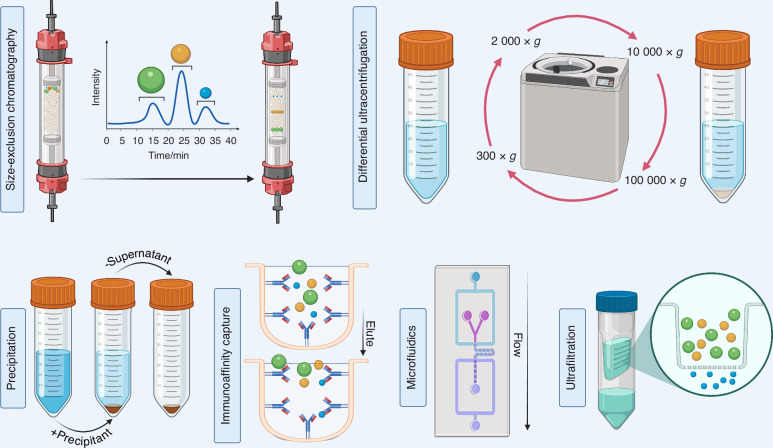
Most utilized exosome isolation approaches. Traditional techniques for exosome isolation comprise differential ultracentrifugation (DUC) and size-exclusion chromatography (SEC). DUC focuses on the separation of exosomes through incrementally increasing centripetal acceleration. SEC employs biofluids as a mobile phase in conjunction with a porous stationary phase, allowing for the differential elution of molecules based on an inverse relationship with their size – specifically, larger particles elute prior to smaller ones, which, upon entering the pores, traverse a longer path resulting in extended elution times. Precipitation utilizes a solution to promote the formation of a polymer-encapsulated vesicle aggregate in substantial quantities. Immunoaffinity capture employs antibodies that target exosomal surface proteins, facilitating the isolation of specific vesicle subpopulations. The microfluidics approach utilizes chips designed for specific antibody-mediated interactions to efficiently capture exosomes. Ultrafiltration operates on the principle of using a filter with a defined pore size, resulting in a vesicle-rich filtrate tailored to the desired dimensions

Currently, there is no one-size-fits-all protocol for the isolation of exosomes;^59^ hence, research on exosomes is significantly hindered by the application of variable extraction/isolation techniques, thereby constraining the understanding of studies focused on exosomes.^60^ Moreover, the heterogeneous protein composition of exosomes complicates their extraction through traditional immune-marking techniques, since not all exosomes exhibit the same classical protein markers, nor are all the recognized markers exclusive to exosomes, because they have also been identified in other categories of EVs. In fact, the ISEV has advocated for a new standardized nomenclature, contingent upon researchers being able to reliably ascertain the endosomal origin of their exosomal preparations. The 2018 MISEV (Minimal Information for Studies of Extracellular Vesicles) recommendations advise the characterization of EVs based on size (i.e., small EVs [sEVs] less than 200 nm; medium/large EVs more than 200 nm), density (i.e., high, medium, low), biochemical composition (i.e., CD63^+^/CD81^+^ EVs), or cellular origin (i.e., hypoxic EVs, oncosomes, and so forth).^61^ Regardless of nomenclature, exosomes and other EVs have been extracted using various methodologies, including microfluidics, immunoaffinity capture (IAC), precipitation, ultrafiltration, SEC, and ultracentrifugation (Fig. 2).^62,63^ The purpose of this review was to examine various techniques employed for the isolation of DSCs-derived exosomes for their applications in regenerative medicine and dentistry. Moreover, we also identified recently optimized methods for efficient isolation of the exosomes, demonstrating improved efficacy over conventional approaches.

## Literature search strategy

An initial search using terms associated with exosomes (i.e., exosomes and extracellular vesicles) and dental stem cells without any limitation of publication year and field of study (medicine, dentistry, etc.) was performed on September 30^th^, 2024. The inclusion criteria comprised original research articles including clinical studies as well as studies conducted in animal or cellular models. Moreover, the included studies examined the therapeutic potential of DSCs-Exos for the treatment of medical and dental diseases. A total of 1 842 publications were retrieved including Elsevier’s Scopus (*n* = 939), Clarivate Analytics’ Web of Science (*n* = 419), and PubMed/MEDLINE (*n* = 484) (Table S1). Of these, 541 were selected and further assessed. Following the full-text screening, a total of 197 research articles were included.

## Literature outcomes of dental stem cells-derived exosome isolation methods

Table S2 lists all 197 studies included in the present review including study references, field or topics covered, and exosomal isolation technique used.^36,45,64–255^

### Overall outcomes of DSCs-Exos isolation methods

**Fig. 3:**
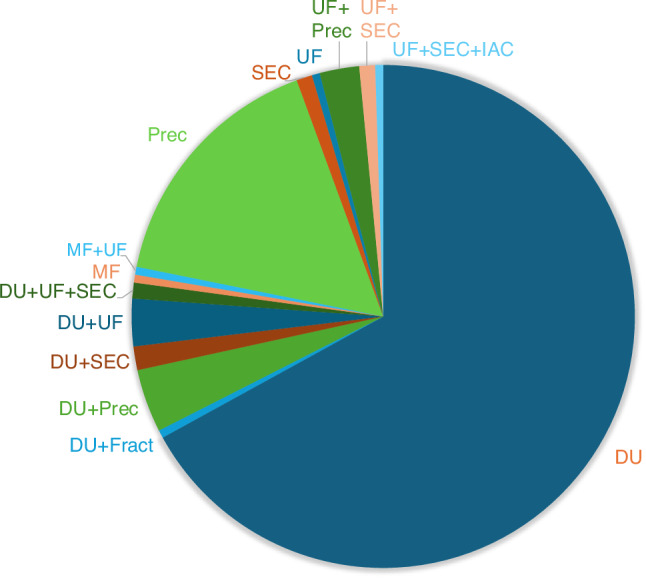
Methods for isolating DSC-Exos used in the 197 included studies

**Fig. 4:**
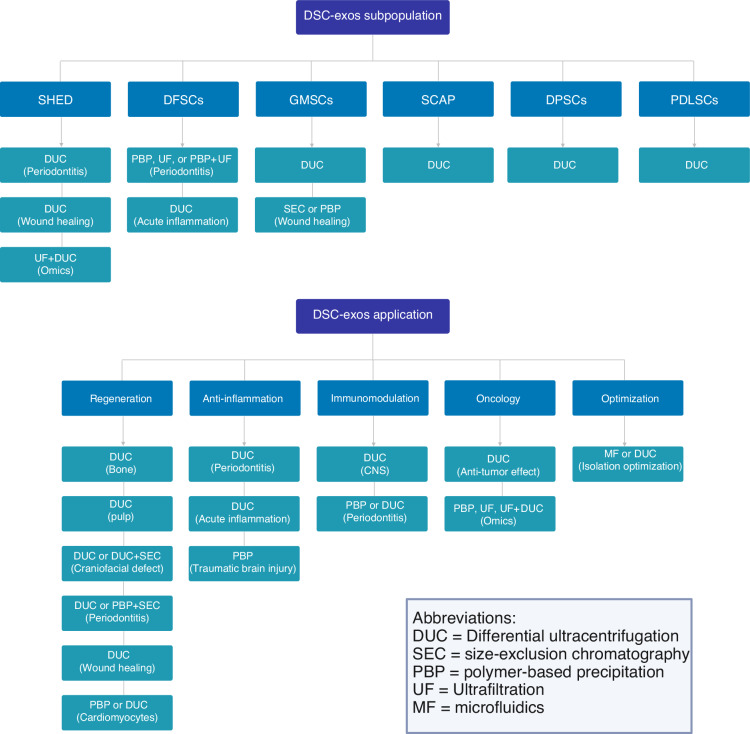
A decision tree depicting the subpopulations and applications of DSC-Exos in different domains of medicine and dentistry utilizing different isolation methods

Overall, in studies investigating exosomes derived from dental cells, a variety of isolation methods have been used, with DUC standing out as the most utilized approach. It was utilized in 132 studies (67%), confirming its position as the gold standard for exosome isolation due to its ability to achieve high-purity exosomes. PBP was the second most frequently used method, reported in 32 studies (16%), offering a cost-effective and accessible alternative. Several studies explored combining DUC with other methods to enhance yield and purity. For instance, DUC combined with PBP was applied in eight studies, with ultrafiltration in six studies, and with SEC in three studies. More complex multi-step approaches, such as the combination of DUC, ultrafiltration, and SEC, were utilized in two studies, indicating a focus on refining the isolation process for greater exosome quality and specificity. Other notable methods included the use of ultrafiltration combined with PBP (*n* = 5), SEC alone (*n* = 2), and microfluidics either as a standalone technique or in combination with ultrafiltration (one study each). Advanced approaches, such as combining ultrafiltration, SEC, and IAC, were explored in one study, reflecting efforts to increase precision in exosome isolation (Fig. 3). Figure 4 summarizes the subpopulations and applications of DSCs-Exos utilizing different isolation methods.

## Recent advancements in dscs-exos isolation

Various isolation methods profoundly influence the yield, purity, and operational efficacy of exosomes.^256–258^ In this regard, Han et al.^259^ investigated the effect of DUC and SEC on the yield of salivary exosomes. They found that salivary exosomes isolated using the SEC approach had a higher yield of particles and particles/protein ratios compared to those isolated utilizing DUC.^259^ Kim et al.^260^ found that the tangential flow filtration (TFF) method showed a remarkable 92.5-fold superiority over ultracentrifugation approach to extract exosomes from umbilical cord mesenchymal stem cells (UCMSCs-Exos), yielding 1.85 × 10^10^ exosomes compared to ultracentrifugation’s 0.02 × 10^10^ for an equivalent cell count.^260^ This substantial scalability renders TFF particularly advantageous for extensive clinical applications. Moreover, employing TFF with optimized serum exosome depletion markedly diminished the presence of impurities (such as low-density lipoprotein [LDL] cholesterol) and augmented MSCs-Exos purity by a factor of 15.6, as verified through CD73 surface marker evaluation. The utilization of a 500 kDa filter enhanced purity relative to a 300 kDa filter by effectively eliminating non-exosomal contaminants.^260^ Additionally, both isolation methods successfully preserved the structural integrity of exosomes (characterized by double-layered spheres via transmission electron microscopy) and maintained tetraspanin markers (CD9, CD63, and CD81).^260^

Hadady and colleagues^71^ in 2022 evaluated two isolation approaches (ultrafiltration and micro-electromechanical systems [MEMS]) for the isolation of SCAPs-Exos. They found that the MEMS approach is capable of isolating a smaller and more effective EVs’ population that safeguards the visual acuity, highlighting the concept that exosomal isolation approaches determine the therapeutic efficacy and the compositions of the isolates. The suggested MEMS apparatus provides adaptable fabrication alternatives, accompanied by the significant benefit of comparatively modest expense. These initiatives are essential for an extensive array of clinical and biomedical applications as an exceptionally practical and essential contender, given that a MEMS device can be utilized to produce therapeutics tailored to individual patients.^71^

The application of alternating current (AC) electrokinetic forces for the manipulation of EVs within a microfluidic device has emerged as a robust and sensitive methodology for diagnostic and trapping applications. Since various AC electrokinetic forces might influence the overall dynamics of particles and fluids within such as device, the electrokinetic confinement of EVs necessitates a comprehensive investigation of all predominant forces at play, contingent upon the specific experimental parameters employed. Hence, Hadady et al.^72^ assessed the isolation of SCAPs-Exos on interdigitated electrode arrays by incorporating two significant AC electrokinetic phenomena, i.e., dielectrophoresis and AC electroosmosis (ACEO). The study revealed that the motion of ACEO fluid significantly influences the localization of SCAPs-Exos in experimental settings. The application of this optimization protocol facilitates a direct correlation with empirical findings, demonstrating a favorable qualitative alignment in behavior. Understanding the underlying physics governing the collection of EVs would aid researchers in manipulating their dynamics within microfluidic systems using AC electrokinetics, thereby advancing the development of innovative research instruments such as biochips and biosensors for exosomal isolation.^72,261^

The culture of three-dimensional (3D) dental pulp pluripotent-like stem cells (DPPSCs) for purposes except for exosome isolation has been documented. Given the benefits of 3D culture conditions for stem cells compared to traditional 2D monolayer systems—particularly regarding in-vivo mimicry, signaling pathways, and improved therapeutic outcomes—it was put forward that exosomes obtained from the 3D culture of these stem cells would similarly demonstrate heightened therapeutic potential for applications in regenerative medicine. Hence, a study by Faruqu et al.^262^ aimed to develop a practicable serum-free 3D culture environment for DPPSCs, facilitating the isolation of exosomes from the stem cells for prospective use in regenerative therapies, while ensuring that the phenotype of the parent cells remains unaltered. They found that a 12-day incubation of DPPSCs in a serum-free 3D culture permitted the isolation of DPPSCs-Exos with minimal contamination from non-exosomal vesicles.^262^ Future studies should aim to investigate the therapeutic potential of DPPSCs-Exos in vitro and within pre-clinical animal models. Additionally, examining the applicability of DPPSCs-Exos in drug delivery systems would be of substantial interest.

In 2024, Duan et al.^45^ presented an innovative approach utilizing ultracentrifugation for obtaining high-purity artificial cell-derived vesicles (ACDV) from DPSCs lysate. A comparative examination was conducted between ACDVs and those obtained via ultracentrifugation. The ACDVs isolated from the cellular lysate conform to the established criteria for EVs and exhibit a comparable protein secretion profile. Furthermore, the novel ACDVs significantly enhanced wound healing, modulated collagen regeneration, and diminished the synthesis of inflammatory mediators akin to EVs. Notably, the isolation efficacy was found to be augmented by 16-fold in comparison to the EVs isolated through the ultracentrifugation method.^45^

According to the International Extracellular Vesicle guidelines,^61^ EV purity is an essential parameter for subsequent analyses and therapeutic applications; nevertheless, current approaches for EV isolation fail to achieve particles of sufficient purity. Hence, Han et al.^161^ found that the purity of sEVs was quantified at 10^8^ sEV particles/μg of protein, which is below the recommended threshold for highly pure EVs (i.e., 3 × 10^10^ EV particles/μg of protein).^257^ The presence of contaminants such as cellular debris, lipids, or proteins in impure EVs can significantly influence their biological functionality, thereby obstructing the precise interpretation of their impacts. Such contaminants might disrupt downstream processes, ultimately undermining effectiveness and leading to erroneous outcomes.^61^ This indicates that further refinement of the sEVs isolation approach is necessary, or that supplementary measures, such as RNase/proteinase/DNase treatments, should be implemented to improve EV purity.^61^ Han et al.^161^ successfully isolated exosomes, microvesicles, and apoptotic bodies from human gingival fibroblasts, human osteoblasts, and human PDLSCs utilizing their in-house SEC for sEV enrichment. Their isolation approach yielded approximately 6 × 10^7^ sEV particles/mL of conditioned media,^161^ which is substantially greater than the previously documented sEV isolation from human PDLSCs via ultracentrifugation, which reported 1.08 × 10^7^ sEV particles/mL of conditioned media^146^ and corroborates the earlier observations that the SEC technique can produce a roughly 5-fold increase in sEV enrichment compared to the ultracentrifugation method.^259^

## Moving beyond traditional methods to isolate dsc-exos

### Limitations of traditional exosome isolation methods

Since DUC was found to be the most widely used method to isolate DSCs-Exos, its inherent limitations, warrant detailed discussion. These limitations include suboptimal yield, variable outcomes, prolonged processing times, the requirement for substantial sample volumes, costly laboratory apparatus, and the necessity for highly skilled personnel.^263,264^ Furthermore, the exceedingly high centrifugal forces can compromise the integrity of exosomes, thereby diminishing the viability of their molecular constituents for subsequent analytical procedures. Crucially, this method is unsuitable for the processing of clinical samples, as it demands sample volumes that significantly exceed those typical of liquid biopsies, is labor intensive ( > 4 h per sample), entails multiple manual operations, and results in diminished exosome yield.^265,266^ Additional constraints pertain to the co-purification of exosomes alongside contaminants including protein aggregates and other particulate matter (i.e., lipoproteins) that exhibit comparable dimensions to exosomes, consequently undermining the precision of downstream analyses, which is essential for clinical applications.^267^

Regarding polymer-based precipitation, precipitation reagents (polyethylene glycol) might contaminate the exosome preparation and interfere with downstream applications such as proteomics or RNA analysis. Moreover, difficulties in the complete removal of the polymer residues can affect exosome characterization and functional studies.^268^ The columns of SEC typically handle small volumes of sample, making the approach unsuitable for large-scale isolation.^269^ Concerning ultrafiltration, filters can become clogged with other EVs, debris, or proteins reducing efficiency and recovery rates. Additionally, exosomes can adhere to the filtration membrane, leading to sample loss and inconsistent yield.^268^ Fractionation, similar to DUC, requires extensive preparation, and its execution is labor-intensive and time-consuming.^270^

### Limitations of label-based exosome isolation approaches

In label-based isolation approaches (i.e., IAC, magnetic bead-based isolation, fluorescence labeling, biotinylated antibody-based isolation, and immunoglobulin-G antibody-based capture), capture molecules (such as aptamers and antibodies) that possess the capacity to physically or chemically bind to specific lipid composition or proteins on the surface of exosomes function as labels.^271–273^ Although physical entanglements and non-covalent interactions, such as hydrophobic interactions, van der Waal forces, ionic bonds, and hydrogen bonds, play a role in the attachment of capture molecules to target exosomes, the predominant labeling strategies rely on covalent interactions (for instance, fluorescent dyes covalently linked to exosomes, or peptides covalently conjugated to exosomes).^274^ Research has demonstrated that immunoaffinity-based microfluidic platforms, such as microplate-based enzyme-linked immunosorbent assays (ELISA), can effectively isolate specific populations of EVs, while minimizing the co-purification of extraneous particles.^275^

The literature suggests that label-based isolation approaches enhance the efficiency of recovering pure exosomes from biological fluids including urine, serum, saliva, and plasma.^276–279^ Despite ensuring the separation purity of labeled exosomes, these strategies require further refinement to achieve higher throughput and to target multiple EV populations utilizing diverse types of labels.^280^ Importantly, these methods incur significant cost due to the expensive labeling protocols. Moreover, they are generally intricate and time-consuming, necessitating complex preparation stages for labeling and additional washing steps to detach the labels from the exosomes. Crucially, capture molecules may inadvertently contaminate the sample and modify exosome characteristics potentially leading to erroneous results during subsequent characterization and undermining the reliability of downstream analyses in the clinical context.^267^

## Potential of label-free approaches for isolation of dsc-exos

### Sieving separation approaches

The sieving separation approach, especially when incorporated into sophisticated microfluidic systems, may present significant possibilities for the extraction of DSCs-Exos. This technique can effectively isolate DSCs-Exos from gingival crevicular fluid (GCF), saliva, or supernatants of various tissues. DSCs-Exos derived from these biological fluids contain molecular indicators (i.e., proteins, miRNAs) that are instrumental for the early detection of periodontal diseases, oral cancer, and other oral and dental diseases. Cutting-edge sieving systems, such as those using TFF, considerably reduce contamination from proteins and debris, hence enhancing the precision of biomarker-focused diagnostics.^281^

Sieving separation can be used to isolate DPSCs-Exos or PDLSCs-Exos for application in regenerative therapies. These exosomes can facilitate osteogenesis, angiogenesis, and tissue repair in periodontal defects and dental implants. Exosomes purified through sieving separation can be incorporated into biomaterial scaffolds to augment their osteoinductive and biocompatibility characteristics.^7^ Moreover, compact sieving devices allow for real-time exosome isolation at the point of care.^282^ This capability is particularly advantageous for monitoring therapeutic responses, such as during periodontal treatment or the integration of implants.

Sieving separation, particularly with ultrafiltration or TFF, is notably more expedient than traditional DUC, significantly decreasing isolation durations while maintaining exosome integrity.^54^ In contrast to DUC, sieving systems apply lower mechanical stress on exosomes, thereby preserving their structural and functional integrity, which is essential for therapeutic applications.^283^ Membrane-based systems are capable of processing diverse sample volumes, rendering them versatile for both research and clinical applications.^284^ Microfluidic sieving systems equipped with adjustable pore sizes facilitate precise exosome separation based on size.^285^ These systems can particularly be effective for isolating specific subpopulations of DSCs-Exos pertinent to dental diseases. TFF inhibits membrane fouling, potentially supporting continuous operation and enhancing recovery rates of DSCs-Exos.^281^ The exosomes isolated using sieving separation can serve as natural carriers for drugs, proteins, or miRNAs.^286^ These DSCs-Exos-based delivery systems could be used to treat dental diseases such as peri-apical infections or periodontitis.

### Electrical separation approaches

The use of electrical separation methods, such as electrophoresis and dielectrophoresis (DEP), presents significant opportunities for isolating EVs,^287^ including DSCs-Exos. These techniques, which exploit the unique physical and electrical characteristics of exosomes,^288^ offer a range of advantages for dental and regenerative applications. Electrical separation methods have the potential to efficiently isolate DSCs-Exos from various biological fluids such as culture media,^289^ saliva, and GCF. Compared to conventional methods such as DUC, this method is particularly critical for dental applications, where intact and functional DSCs-Exos are required.

Microfluidic platforms that incorporate electrical separation mechanisms provide scalable and automated solutions for exosome isolation.^290^ These systems, which integrate microelectrodes or nanoporous membranes, are highly efficient for processing small sample volumes,^287^ such as those obtained from dental tissue fluids or culture media. Automated systems could facilitate high-throughput exosome isolation,^263^ making them practical for regenerative dentistry procedures, such as the preparation of bioengineered scaffolds or injectable therapeutic formulations. Achieving high purity is critical for clinical-grade exosome preparations.^291^ Electrical separation techniques are well-suited for this purpose (1) DEP-based isolation exploits differences in dielectric properties to improve the separation of DSCs-Exos from contaminants including protein aggregates or other nanoparticles;^292^ and (2) combined approaches, such as electrophoresis with membrane filtration,^293^ can further enhance the purity of DSCs-Exos obtained from dental culture supernatants.

Electrical separation approaches can facilitate the isolation of exosomes for incorporation into dental biomaterials. Isolated DSCs-Exos can be incorporated into hydrogels, scaffolds, or bone graft materials to enhance periodontal and alveolar bone regeneration. The gentle isolation process preserves exosome bioactivity,^287^ ensuring their therapeutic potential when integrated with biomaterials. DSCs-Exos can also be isolated from readily available fluids such as saliva or GCF, which are rich in biological information. Electrical separation methods are particularly suited for these applications (1) electrophoresis can exploit the negative charge of exosomes for efficient separation;^294^ and (2) DEP offers the capability to isolate exosomes rapidly, even in complex biological fluids, through precise control of electric fields.^295^

### Inertial separation approaches

Inertial microfluidics may represent a cutting-edge platform for the isolation of DSCs-Exos. Leveraging size-dependent hydrodynamic forces, including inertial lift, viscous drag, and Dean vortices, these systems offer a precise and scalable approach to separate nanoscale EVs from complex biological fluids.^296,297^ Inertial separation systems provide a label-free method for isolating EVs,^298^ including DSC-Exos, from biofluids such as dental pulp culture media, saliva, and GCF. Spiral microchannels generate Dean vortices, directing smaller particles such as exosomes toward the inner channel wall while larger particles such as debris and cells are displaced outward.^299^ This enables high-throughput, single-step separation. The ability to continuously process biofluids makes these systems well-suited for both research and clinical applications,^300^ where rapid DSCs-Exos isolation is critical. The minimal mechanical stress in these systems ensures the preservation of DSCs-Exos integrity, including their bioactive components such as proteins, miRNAs, and cytokines.

The flexibility of inertial microfluidics to handle biofluids with varying viscosities can ensure compatibility with small-volume samples^301^ collected during dental procedures. These designs generate stronger Dean vortices compared to rectangular geometries,^302^ improving the lateral displacement of DSCs-Exos and enhancing separation efficiency. Optimized channel dimensions allow for higher sample processing rates, supporting clinical workflows and large-scale research studies.^303^

### Viscoelastic separation approaches

Viscoelastic separation is a label-free method that exploits the elastic lift forces acting on particles of varying sizes within a viscoelastic fluid medium.^304^ Unlike affinity-based isolation techniques that rely on capture molecules such as antibodies or aptamers,^305^ this method preserves the native properties of exosomes, which is essential for ensuring their functional integrity in clinical applications. The viscoelastic separation process allows for the continuous and efficient fractionation of exosomes from larger EVs such as microvesicles and apoptotic bodies.^306^ This size-dependent separation is achieved by exploiting the differential lateral migration of particles in viscoelastic flow.^305,307^ Isolating purified exosome subpopulations from DSCs-Exos enhances the reproducibility and efficacy of their downstream therapeutic applications, particularly in dental tissue repair.

Viscoelastic microfluidic systems are capable of processing sample volumes at flow rates exceeding 1 mL/min,^308^ offering a significant advantage over conventional isolation methods such as DUC or polymer-based precipitation. This high-throughput capability is particularly relevant for clinical-scale applications, where large quantities of EVs are required. Stem cell cultures or tissue samples, such as dental pulp or periodontal ligament tissue, typically yield substantial EV quantities, and viscoelastic separation ensures efficient processing to achieve sufficient exosome yields. The performance of viscoelastic separation systems can be tuned by adjusting parameters such as the viscoelastic fluid composition, channel geometry, and flow rate.^309^ This flexibility allows for the optimization of isolation conditions specific to DSCs-Exos, ensuring effective separation from complex biological matrices such as stem cell culture supernatants or dental tissue extracts. Moreover, advancements in microchannel design, including wavy or curved geometries, can further enhance particle focusing and separation efficiency.^310^

### Deterministic lateral displacement sorting

The application of deterministic lateral displacement (DLD) technology for isolating DSCs-Exos holds significant promise due to its ability to separate nanoparticles with high precision. DLD technology separates particles based on size,^311^ making it particularly suited for isolating exosomes from complex biological fluids such as dental tissue lysates. By tailoring the gap size, displacement angle, and total array length of the DLD device,^312^ it is possible to selectively isolate exosomes while removing larger particles such as apoptotic bodies, microvesicles, and debris. Dental tissue samples can be collected through minimally invasive procedures. The incorporation of DLD systems allows for streamlined processing of small volumes of fluid which is advantageous for dental applications where sample availability may be limited. DLD platforms are scalable, with parallelized nano-DLD arrays capable of processing larger sample volumes efficiently.^313^ For clinical applications, this scalability is critical for rapid and effective exosome isolation, addressing the time-sensitive nature of dental treatments.

### Field flow fractionation

Field flow fractionation (FFF) is a sophisticated, flow-based separation technique^314^ with significant promise for isolating DSCs-Exos. FFF is well-suited for separating nanoparticles based on their biophysical properties such as size, density, and surface charge.^315^ By fine-tuning operational parameters—such as channel height, flow velocity, and applied external fields—FFF can effectively isolate exosome populations from complex biological fluids.^314^ This allows for the separation of pure exosome populations, free from other EVs and contaminants. In contrast to conventional DUC, FFF employs gentle hydrodynamic forces during separation.^316^ This approach preserves exosome morphology and bioactivity, ensuring their suitability for therapeutic applications in regenerative dentistry.

Advanced FFF modalities, such as asymmetrical flow FFF (AF4) and electrical FFF, enable the fractionation of exosome subpopulations based on subtle differences in size, surface charge, and density.^317^ For example, FFF can separate subpopulations of small (~60–80 nm) and large (~90–120 nm) exosomes, as well as exomeres (~35 nm), which may exhibit unique biological properties.^318,319^ This capability facilitates targeted investigations into the functional roles of different exosome subsets and their specific applications in dental tissue regeneration. FFF systems can be integrated with analytical detection methods, such as ultraviolet-visible spectroscopy, multiangle light scattering, and nanoparticle tracking analysis.^320–322^ These combined platforms provide comprehensive data on exosome size, concentration, and surface properties, ensuring high-quality exosome characterization.^322^ This integration is crucial for standardizing exosome preparations for therapeutic applications in dentistry.

### Pinched flow fractionation

Pinched flow fractionation (PFF) is a microfluidic-based size-sorting technology that has demonstrated significant promise for the isolation of exosomes.^323^ Given its precise separation capabilities, PFF offers a valuable tool for isolating DSCs-Exos. PFF facilitates the efficient separation of particles based on size by leveraging laminar flow dynamics in a microchannel with a pinched and broadened segment.^324^ This technology is particularly effective for isolating exosomes from larger EVs, including apoptotic bodies and microvesicles.^267^ By aligning particles to the sidewall in the pinched segment and directing them along distinct streamlines based on size, PFF ensures high-resolution separation of pure exosome populations.^325^ This capability is critical for isolating DSCs-Exos for dental tissue regeneration.

PFF systems are capable of processing large sample volumes with high throughput. For example, recent studies demonstrated PFF devices capable of achieving flow rates up to 200 μL/min while maintaining separation efficiency.^326^ This scalability makes PFF suitable for processing clinical samples, enabling routine isolation of DSCs-Exos in dental research and practice. The size-specific separation capability of PFF allows for the fractionation of distinct exosome subpopulations. This feature is particularly advantageous for isolating exosome subsets with unique therapeutic potential.^327^ Small exosomes ( ~ 30 to 80 nm) may exhibit enhanced pro-angiogenic and neuroprotective properties beneficial for dental pulp and nerve regeneration. Large exosomes ( ~ 90 to 150 nm) may be enriched with osteogenic factors relevant to alveolar bone repair and periodontal ligament regeneration.

The microfluidic nature of PFF enables seamless integration with real-time analytical tools such as nanoparticle tracking analysis, fluorescence microscopy, and dynamic light scattering.^328^ This integration allows for immediate characterization of exosome size, concentration, and purity,^329^ enabling the development of standardized workflows for exosome isolation and quality control in dental research and therapeutic applications. The design of PFF devices can be optimized for specific dental applications. Adjustments in microchannel width, pinched segment dimensions, and outlet configurations allow for the fine-tuning of particle separation resolution.^330^ For instance, narrower pinched segments improve the isolation of smaller exosomes, while asymmetrically arranged outlets enhance separation precision and recovery of distinct exosome subpopulations.^331^ Such customization enables efficient isolation of exosomes tailored to therapeutic needs in dentistry.

### Acoustic separation approaches

Acoustic separation is an innovative, label-free method for isolating exosomes with high precision and minimal damage to their structural and functional properties.^332^ By leveraging acoustic wave technology within microfluidic platforms, this approach offers significant advantages for isolating DSCs-Exos. Acoustic separation enables the efficient isolation of exosomes by exploiting differences in physical properties such as size, density, and compressibility.^333^ Larger EVs (e.g., microvesicles) are directed toward acoustic pressure nodes under the influence of acoustic radiation forces, whereas smaller exosomes remain confined in the central flow.^334^

Acoustic microfluidic devices are capable of continuous-flow processing,^285^ allowing for rapid and efficient isolation of DSCs-Exos from cell culture supernatants or biological fluids. The high throughput and minimal sample loss make acoustic separation highly suitable for clinical-scale exosome production, addressing the growing demand for exosome-based therapeutics in dentistry. Acoustic separation technology can be fine-tuned to selectively isolate exosome subpopulations based on unique biophysical properties^335^ (1) variations in membrane cholesterol content or lipid composition influence the acoustic contrast factor of exosomes,^336^ enabling their selective isolation for specific regenerative applications; and (2) By optimizing parameters such as acoustic wave frequency, amplitude, and channel dimensions,^337^ specific exosome subpopulations can be isolated to enhance angiogenesis, osteogenesis, or immunomodulation as required for dental applications.

Acoustic separation enables efficient discrimination between exosomes and impurities such as lipoproteins and protein aggregates, which often have overlapping physical properties. By optimizing the acoustic field parameters, high-purity exosome fractions can be obtained, reducing the risk of contamination and enhancing the safety of clinical applications in dentistry. The microfluidic devices used in acoustic separation can be tailored to meet the specific requirements of dental research and practice. Adjustments in transducer geometry, channel dimensions, and flow patterns allow for optimized isolation of DSCs-Exos, enabling precision-based therapeutic applications. Table S3 summarizes the abovementioned label-free isolation approaches with a set of their outcomes. DUC has also been included in Table S3 to enable a comparison of this frequently utilized method to report label-free exosome isolation techniques.^48,55,305,314,338–361^

While various label-free exosome isolation techniques have been extensively studied in the broader field of EVs’ research, their specific application to DSCs-Exos remains largely unexplored and unverified. The authors believe that these isolation approaches successfully applied to non-DSCs-Exos hold significant potential for adaptation to DSCs-Exos. This highlights an important area for future investigations to optimize and standardize DSCs-Exos isolation methods.

## Discussion

This benchmark study aimed to provide a comprehensive review of 197 studies regarding the methodologies utilized to isolate exosomes derived from DSCs as well as their potential applications in medical and dental fields. A clear trend emerged in the literature,^47,62,296,362–368^ highlighting differential adoption of isolation methods based on study focus and cell type. This study confirmed that DUC remains the dominant method across various research areas, maintaining its role as the conventional approach. For exosomes derived from SCAPs, DFSCs, GMSCs, SHEDs, PDLSCs, DPSCs, and ABMSCs, DUC was used in 52% – 82% of the studies, with popularity varying slightly across cell types. Notably, DUC’s dominance was observed particularly in studies emphasizing high-purity exosome isolation for regenerative applications.

Other approaches such as precipitation, ultrafiltration, and SEC were frequently applied alongside DUC to enhance exosome yield, purity, and scalability. Specifically, Moreover, precipitation method showed cost-effectiveness and scalability, making it a practical alternative, especially for GMSCs, PDLSCs, and DPSCs. Microfluidic and hybrid approaches, though less common, indicated a shift towards more precise and scalable isolation techniques, with studies increasingly testing their efficacy across multiple DSCs-Exos applications. Overall, an evolving trend emerged toward optimizing DSCs-Exos isolation for specific clinical applications, balancing purity, yield, and feasibility.

The widespread utilization of DUC was evident across various medical and dental fields, particularly in studies related to periodontitis, dental pulp regeneration, CBDs, diabetes, and CNS disorders. Periodontitis consistently appeared as a major focus, underscoring the demand for high-purity exosomes to investigate inflammatory and regenerative pathways. DUC, which relies on repeated high-speed centrifugation to separate exosomes by density,^46^ was particularly suited for applications in cell-derived biomaterials and regenerative medicine due to its ability to preserve exosome bioactivity without introducing chemical reagents.^59,369^

Precipitation emerged as a preferred method for studies requiring large exosome quantities, particularly in CBDs, ischemic heart disease, periodontitis, dental pulp regeneration, and immunomodulation applications. This method, which relies on polymer-based precipitation,^370–372^ was advantageous in studies requiring substantial exosome volumes to achieve therapeutic impact. It was frequently paired with DUC, especially in cancer and tissue regeneration studies, where a two-step process was used to ensure both high yield and enhanced purity. In cancer research, where exosome content is analyzed for biomarkers or therapeutic targeting, this hybrid approach ensured that exosomes were abundant yet free from contaminants. Similarly, in tissue regeneration, purified exosomes with retained bioactivity were crucial for promoting cellular repair and differentiation.

Ultrafiltration and precipitation methods were applied mainly in periodontal regeneration studies, often in combination with DUC to enhance specificity in exosome isolation. Ultrafiltration, which uses membrane-based size-selective filtering, was particularly effective in maintaining exosome integrity and functionality – critical for modulating inflammation and promoting periodontal tissue healing.

A more complex methodological landscape was observed in studies focusing on tissue regeneration, CNS diseases, and myocardial infarction, where DUC, ultrafiltration, and SEC were used in combination to maximize isolation efficiency. SEC, although less commonly used alone, was notable in diabetes, hyperthermia, and cancer studies, where high specificity in molecular differentiation is required.

Studies on DSCs-Exos demonstrated a broad range of applications across medicine and dentistry, reflecting a growing interest in their regenerative and therapeutic potential. Among the most frequently researched areas, periodontal regeneration (20%)^64,81,88,109,146,181^ emerged as a dominant focus, reinforcing the strong emphasis on using DCSs-Exos to promote periodontal tissue healing and regeneration. CBDs (13%)^36,74,108,120,153,155,180,185,254^ and dental pulp regeneration (13%)^65,79,136,143,182,186^ also received significant attention, highlighting the role of DSCs-Exos in hard and soft tissue repair within the oral and maxillofacial regions. Similarly, CNS disorders (6%)^78,115,124,127,138,145,198,208^ represented a key area of investigation, underscoring the expanding interest in DSCs-Exos-mediated neuroprotection and neural tissue repair.

Beyond regenerative applications, the research landscape reflected an increasing focus on refining DSCs-Exos isolation and functional enhancement. Studies on optimization techniques (4%)^71,72,86,87,97,161,193^ and tissue regeneration (4%)^70,103,204^ pointed to ongoing efforts to improve the exosome efficacy for therapeutic applications. In parallel, cancer research (3%)^151,184,191,225^ explored DSCs-Exos’ role in tumor progression, immune modulation, and potential biomarker discovery. Furthermore, studies on arthritis (3%),^98,101,102,125,219,224^ coronary artery disease (3%),^215^ diabetes (3%),^68,174^ and immunomodulation (3%)^199,206,216,222^ demonstrated the broadening of DSCs-Exos research into systemic and inflammatory diseases.

Research on OTM^147,149,159^ and omics profiling^96,110,111,229^ indicated a growing interest in DSXs-Exos-based precision medicine, leveraging exosomal content for diagnostic and therapeutic advancements. Investigations into Sjögren’s syndrome,^122,131,192,239^ wound healing,^92,95,116,134,220^ retinal degeneration,^71,87^ sciatic nerve injuries,^104,211,236^ and tooth avulsion^66,144,189^ (each < 2% suggested early-stage exploration into specialized applications, with potential for future translation into clinical practice. Additionally, studies on multiple sclerosis (1%),^170,171^ osteoporosis (1%),^140,255^ Parkinson’s disease (1%),^129,141^ salivary gland function (1%),^178,179^ and TMJ disorders (1%)^83,139^ reflected the expanding interdisciplinary interest in DSCs-Exos-mediated therapeutics. Finally, individual studies examined DSCs-Exos applications in acute renal injury,^73^ apical periodontitis,^77^ dental caries,^168^ dental implants,^231^ systemic lupus erythematosus,^130^ and trigeminal neuralgia,^135^ reinforcing the versatility of DSCs-Exos in diverse clinical contexts.

The paucity of comparative investigations evaluating DSCs-Exos and MSCs-Exos has been observed in the literature. While exosome-based regenerative strategies have been widely explored, direct comparisons between these two sources of exosomes remain insufficiently characterized. This lack of data hinders a comprehensive understanding of their distinct molecular compositions, bioactive cargo, and functional properties, limiting the ability to discern their relative advantages and therapeutic potential. Future studies should focus on systematically comparing the proteomics, transcriptomics, lipidomics, and metabolomics profiles of these exosomes, as well as their respective roles in key biological processes such as osteogenesis, angiogenesis, and immunomodulation. Addressing this knowledge gap through well-designed comparative analyses will provide valuable insights into the translational applications of DSCs-Exos and their potential superiority or complementarity to MSCs-Exos in regenerative medicine.

While this review has provided an overview of recent advancements in exosome isolation, the studies primarily focused on exosomes derived from SCAPs, PDLSCs, and DPSCs. This narrow focus may limit the comprehensiveness of this review in capturing the full potential of DSCs-Exos in regenerative applications. Despite the increasing interest in exosome-based therapies, there remains a paucity of research investigating newer isolation methods for exosomes from other DSCs, such as GMSCs, ABMSCs, SHEDs, and DFSCs. The absence of studies evaluating their novel isolation methods restricts our ability to provide a more inclusive and critical discussion. Future research should aim to explore novel exosome isolation strategies across a broader range of DSCs, enabling a more complete comprehension of their yield, purity, and functionalities.

## Roadmap for dsc-exos isolation

The increased focus on the significance of DSCs-Exos in biomedical research has underscored the urgent requirement for innovative approaches aimed at the swift and precise isolation of DSCs-Exos while minimizing the need for extensive sample preparation. In this discourse, the authors outline the following critical domains for prospective advancements:

### Characterization and biological validation

Presently, the approaches employed for quantifying the precise or optimal yield of DSCs-Exos remain predominantly semi-quantitative. There exists a significant demand for both qualitative and quantitative evaluations of DSCs-Exos, alongside the establishment of unequivocal biological controls.^373^ It is crucial to acknowledge that there is no universally applicable solution for the characterization of DSCs-Exos. The characterization will be contingent upon the specific clinical application. Incorporating labeled DSCs-Exos into experiments provides a dependable approach for determining yield, whether through quantitative reverse transcription polymerase chain reaction (qRT-PCR) or fluorescence measurement. Additionally, it is advisable to evaluate not only the yield (i.e., presence of selected markers) but also the specificity (i.e., absence of contaminants).^373^

### Specificity and reproducibility

Devices equipped with on-chip technologies, such as protein-specific markers for sorting exosomes, may enable clinicians to access a pertinent subpopulation of exosomes relevant to their site of interest. The prevailing methodologies for isolation have not effectively ensured reproducibility in DSCs-Exos recovery, which can be attributed to inconsistencies in sample handling. Ideally, the emerging approaches such as label-free isolation, should undergo testing across various laboratories to evaluate their reproducibility and consistency, thereby demonstrating standardized performance.

### Overcoming potential limitations of emerging label-free isolation approaches

In this review, most of the included studies have employed a singular isolation approach and are not devoid of challenges. Future label-free approaches might therefore benefit from hybrid methodologies that integrate two or more isolation techniques; for instance, an initial separation based on size could eliminate larger cells from dental tissue samples, followed by an immunological separation to access a specific subpopulation of exosome.

The primary challenge, as with all label-free approaches, is to deliver a solution to a real clinical question. Two distinct pathways are likely to develop:(1) to investigate exosome biology within the research domain, necessitating refined isolation techniques. For this, the benefit of label-free approaches will lie in providing a more economical alternative to DUC with minimal capital investment; and (2) concentrating on creating tools to enhance liquid biopsy preparation for next-generation sequencing. This difficulty will be in the swift extraction from complex biofluids. To integrate seamlessly into clinical settings, the approaches will need to be more robust and cost-effective compared to their existing counterparts. The developmental challenge will encompass (a) lowering approach costs, a factor that must be considered during the design phase, potentially involving the removal of the need for structures smaller than 80 μm, at this point, photolithography could be supplanted by rapid throughput and high-volume manufacturing processes; and (b) furthermore, achieving the appropriate throughput will be imperative, which might necessitate multiple units within a single device.

### From laboratory to clinical settings

DUC is presently employed in research environments, yet it has not been widely adopted in clinical settings. Should a DSCs-Exos-based approach be implemented in real-world clinical settings, one might envision that in non-emergent scenarios, such as oral cancer diagnosis, the utilization of a centralized laboratory equipped with DUC technology could be viable, contingent upon the reproducibility of the isolation process. However, if standardized efficacy is established using label-free approaches, it would facilitate the development of more rapid, dependable, and economically viable DSCs-Exos-based interventions. Moreover, this advancement could permit enhanced analysis of DSCs-Exos, given that research has demonstrated a significant reduction in DSCs-Exos quantity from biofluids even when preserved at – 80 °C^374^ and within two hours.^375^ For DSCs-Exos application in clinical settings, the approach should ideally enable the clinician to swiftly and reliably isolate DSCs-Exos from biofluids with a 30-minute interval post-sample collection.

## Conclusion

This benchmark analysis of exosomes highlights DUC as the primary method for isolating exosomes from diverse DSC sources, demonstrating its effectiveness in producing high yields while preserving exosomal structure and bioactivity. To further improve exosome purity, DUC was often used alongside precipitation and ultrafiltration techniques, especially in research areas requiring high exosome volume and specificity. The emergence of advanced methods, such as MEMS, signifies progress toward scalable and cost-efficient isolation strategies, highlighting a trend toward achieving the high-purity exosome preparations essential for therapeutic efficacy. This study underscores the critical need for continued refinement in exosome isolation techniques to support the purity and functionality of exosomal preparations, which is essential to advancing their application across regenerative, diagnostic, and therapeutic platforms in both dental and broader medical contexts.

## Supplementary information

Supplemental Table S1

Supplemental Table S2

Supplemental Table S3

## Data Availability

Data available on request from the corresponding author.
