# Supplementary material for: Isolation methods of exosomes derived from dental stem cells
Source: Int J Oral Sci. 2025 Jun 16;17:50. doi: 10.1038/s41368-025-00370-y (PMC12170887; doi:10.1038/s41368-025-00370-y)
Supplement: Supplementary file 1 — Supplemental Table S1 [file 41368_2025_370_MOESM1_ESM.docx]

**Table S1**. Literature search strategy.

| **Search term (Title, Abstract, and Keywords)** | **Scopus** | **Web of Science** | **PubMed** | **Total** |
| --- | --- | --- | --- | --- |
| “Exosom*” AND “Apical Papilla” AND “Stem Cell*” | 31 | 15 | 16 | 62 |
| “Extracellular Vesicle*” AND “Apical Papilla” AND “Stem Cell*” | 16 | 8 | 11 | 35 |
| “Exosom*” AND “Dental Follicle” AND “Stem Cell*” | 19 | 4 | 4 | 27 |
| “Extracellular Vesicle*” AND “Dental Follicle” AND “Stem Cell*” | 17 | 8 | 9 | 34 |
| “Exosom*” AND “Gingival Mesenchymal” AND “Stem Cell*” | 36 | 13 | 16 | 65 |
| “Extracellular Vesicle*” AND “Gingival Mesenchymal” AND “Stem Cell*” | 21 | 12 | 17 | 50 |
| “Exosom*” AND “Exfoliated” OR “SHED” AND “Stem Cell*” | 65 | 35 | 38 | 138 |
| “Extracellular Vesicle*” AND “Exfoliated” OR “SHED” AND “Stem Cell*” | 28 | 23 | 25 | 76 |
| “Exosom*” AND “Periodontal Ligament” AND “Stem Cell*” | 155 | 62 | 64 | 281 |
| “Extracellular Vesicle*” AND “Periodontal Ligament” AND “Stem Cell*” | 74 | 37 | 45 | 156 |
| “Exosom*” AND “Dental Pulp” AND “Stem Cell*” | 246 | 84 | 101 | 431 |
| “Extracellular Vesicle*” AND “Dental Pulp” AND “Stem Cell*” | 110 | 66 | 78 | 254 |
| “Exosom*” AND “Jaw” AND “Stem Cell*” | 5 | 2 | 3 | 10 |
| “Extracellular Vesicle*” AND “Jaw” AND “Stem Cell*” | 1 | 0 | 1 | 2 |
| “Exosom*” AND “Alveolar Bone” AND “Stem Cell*” | 75 | 32 | 31 | 138 |
| “Extracellular Vesicle*” AND “Alveolar Bone” AND “Stem Cell*” | 40 | 18 | 25 | 83 |
| **Total** | **939** | **419** | **484** | **1842** |
