# Supplementary material for: Isolation methods of exosomes derived from dental stem cells
Source: Int J Oral Sci. 2025 Jun 16;17:50. doi: 10.1038/s41368-025-00370-y (PMC12170887; doi:10.1038/s41368-025-00370-y)
Supplement: Supplementary file 2 — Supplemental Table S2 [file 41368_2025_370_MOESM2_ESM.docx]

**Table S2**. Studies assessing the applications of exosomes derived from dental stem cells with a particular focus on isolation methods.

| **STUDY** | **TOPIC/FIELD** | **ISOLATION METHOD** |
| --- | --- | --- |
| **APICAL PAPILLA STEM CELLS-DERIVED EXOSOMES** | | |
| (Zhang et al. 2023) | Periodontitis (anti-inflammation) | Differential ultracentrifugation |
| (Liu et al. 2023a) | Dental pulp (regeneration) | Differential ultracentrifugation |
| (Lin et al. 2023) | Wound healing in tooth avulsion (regeneration) | Fractionation & differential ultracentrifugation |
| (Nie et al. 2024) | Non-alcoholic steatohepatitis (anti-inflammation) | Differential ultracentrifugation |
| (Jing et al. 2022) | Diabetes (bone regeneration) | Differential ultracentrifugation |
| (Yu et al. 2022a) | Pulpitis (anti-inflammation) | Differential ultracentrifugation |
| (Wang et al. 2020a) | Tissue regeneration and immune regulation | Precipitation |
| (Hadady et al. 2022) | Retinal degeneration (neuroprotective/anti-inflammatory effects; optimization) | Micro-fluidic & ultrafiltration |
| (Hadady et al. 2021) | Optimization | Micro-fluidic |
| (Huang et al. 2022b) | Acute renal injury (anti-inflammatory) | Differential ultracentrifugation |
| (Liu et al. 2020b) | Craniofacial regeneration | Differential ultracentrifugation |
| (Zhuang et al. 2020) | Dentine-pulp complex regeneration | Differential ultracentrifugation |
| (Wang et al. 2019) | Dental pulp (regeneration) | Differential ultracentrifugation |
| (Yang et al. 2022) | Apical periodontitis (regeneration) | Differential ultracentrifugation |
| (Gratpain et al. 2024) | CNS (antiinflammation) | Differential ultracentrifugation, ultrafiltration & size-exclusion chromatography |
| **DENTAL FOLLICLE STEM CELLS-DERIVED EXOSOMES** | | |
| (Li et al. 2024) | Dental pulp (regeneration) | Differential ultracentrifugation |
| (Fu et al. 2023a) | PDL-like tissue regeneration | Differential ultracentrifugation & precipitation |
| (Liang et al. 2024a) | Periodontitis (regeneration) | Ultrafiltration, size-exclusion chromatography & immune-affinity-based capture |
| (Huang et al. 2022c) | Periodontitis (regeneration) | Ultrafiltration & precipitation |
| (Mao et al. 2023) | TMJ osteoarthritis (regeneration) | Ultrafiltration & precipitation |
| (Ma et al. 2022) | Periodontitis (regeneration) | Ultrafiltration & precipitation |
| (Yi et al. 2022) | Periodontitis (regeneration) | Differential ultracentrifugation & ultrafiltration |
| **GINGIVAL MESENCHYMAL STEM CELLS-DERIVED EXOSOMES** | | |
| (Deng et al. 2024) | Optimization | Differential ultracentrifugation |
| (Yu et al. 2022b) | Retinal degeneration (neuroprotective/anti-inflammatory effects; optimization) | Differential ultracentrifugation |
| (Zarubova et al. 2022) | Periodontitis (regeneration) | Differential ultracentrifugation |
| (Liang et al. 2023) | Non-small cell lung cancer (antitumor effect) | Differential ultracentrifugation |
| (Hu et al. 2023b) | Periodontitis (regeneration) | Differential ultracentrifugation |
| (Zeng et al. 2022b) | Tracheal defect (regeneration) | Differential ultracentrifugation |
| (Liu et al. 2023c) | Diabetic wound healing | Differential ultracentrifugation |
| (Della Rocca et al. 2024b) | Acute hypoxia (regeneration of cardiomyocytes) | Precipitation |
| (Sun et al. 2022) | Periodontitis (regeneration) | Differential ultracentrifugation |
| (Shi et al. 2017) | Diabetic wound healing | Size-exclusion chromatography |
| (Silvestro et al. 2020) | Transcriptomics profiling | Precipitation |
| (Della Rocca et al. 2024a) | Optimization (tissue regeneration) | Precipitation |
| (Tian et al. 2022) | Rheumatoid arthritis (anti-inflammatory) | Differential ultracentrifugation |
| (Wang et al. 2020c) | Periodontitis (immunomodulation) | Differential ultracentrifugation |
| (Zhang et al. 2021) | Periodontitis (anti-inflammatory) | Differential ultracentrifugation |
| (Chen et al. 2024a) | Rheumatoid arthritis (immunomodulation) | Differential ultracentrifugation |
| (Bruckner et al. 2023) | Rheumatoid arthritis (immunomodulation) | Differential ultracentrifugation |
| (Diomede et al. 2018b) | Bone tissue regeneration | Precipitation |
| (Rao et al. 2019) | Sciatic nerve regeneration | Differential ultracentrifugation & precipitation |
| (Zhang et al. 2019) | Taste bud regeneration | Precipitation |
| (Wang et al. 2023c) | Bone defect (regeneration) | Differential ultracentrifugation |
| (Della Rocca et al. 2022) | Cardiomyocytes acute hypoxia | Precipitation |
| (Giuliani et al. 2020) | Bone defect (regeneration) | Precipitation |
| **STEM CELLS FROM HUMAN EXFOLIATED DECIDUOUS TEETH-DERIVED EXOSOMES** | | |
| (Wang et al. 2023b) | Periodontal disease (regeneration) | Precipitation |
| (Gao et al. 2022) | Proteomics (regeneration) | Ultracentrifugation & ultrafiltration |
| (Liu et al. 2024) | Osteonecrosis of femoral head transcriptomics (regeneration) | Differential ultracentrifugation |
| (Zheng et al. 2023b) | Periodontitis (regeneration) | Differential ultracentrifugation |
| (Fallah et al. 2024) | Anti-inflammatory | Precipitation |
| (Jin et al. 2023) | Aging tendon (regeneration) | Ultracentrifugation & ultrafiltration |
| (Li et al. 2022c) | Traumatic brain injury | Precipitation |
| (Bastidas et al. 2023) | Re-epithelialization/wound healing (regeneration) | Differential ultracentrifugation |
| (Yu et al. 2024) | Periodontitis (regeneration) | Differential ultracentrifugation |
| (Wu et al. 2019) | Periodontitis (regeneration) | Differential ultracentrifugation |
| (Jing et al. 2024) | Ischemic retinopathy (regeneration) | Differential ultracentrifugation |
| (Guo et al. 2022) | Craniofacial bone defect (regeneration) | Differential ultracentrifugation |
| (Sunartvanichkul et al. 2023) | Diabetic wound healing | Differential ultracentrifugation |
| (Du et al. 2023) | Sjögren’s syndrome (Immunomodulation) | Differential ultracentrifugation |
| (Katahira et al. 2023) | Pressure ulcers (regeneration) | Differential ultracentrifugation |
| (Jonavičė et al. 2021) | Microglia/CNS (immunomodulation) | Differential ultracentrifugation |
| (Lin et al. 2024a) | Osteoarthritis (anti-inflammation) | Differential ultracentrifugation |
| (Koga and Horiguchi 2022) | Erectile dysfunction (regeneration) | Differential ultracentrifugation |
| (Jonavičė et al. 2019) | Microglia/CNS (immunomodulation) | Differential ultracentrifugation |
| (Wang et al. 2020b) | Periodontal disease (regeneration) | Differential ultracentrifugation |
| (Jarmalavičiūtė et al. 2015) | Parkinsonian neurodegeneration (regeneration) | Differential ultracentrifugation |
| (Sonoda et al. 2021) | Systemic lupus erythematosus (immunomodulation) | Differential ultracentrifugation |
| (Chu et al. 2024) | Sjögren’s syndrome (Immunomodulation) | Differential ultracentrifugation |
| (Brunello et al. 2022) | Craniofacial defects (regeneration) | Differential ultracentrifugation |
| (Wei et al. 2020) | Bone defects (regeneration) | Differential ultracentrifugation |
| (Pivoraitė et al. 2015) | Acute inflammation (anti-inflammation) | Differential ultracentrifugation |
| (Xie et al. 2022) | Cutaneous wound healing (regeneration) | Differential ultracentrifugation |
| (Guo et al. 2023) | Trigeminal neuralgia (immunomodulation) | Differential ultracentrifugation |
| (Lu et al. 2024) | Pulp necrosis (regeneration) | Differential ultracentrifugation |
| (Peng et al. 2024) | Mitochondrial dynamics (immunomodulation) | Differential ultracentrifugation |
| (Li et al. 2017) | Traumatic brain injury/microglia/CNS (anti-inflammation) | Precipitation |
| (Luo et al. 2019) | TMJ (anti-inflammation) | Differential ultracentrifugation |
| (Sonoda et al. 2020) | Osteoporosis (immunomodulation) | Differential ultracentrifugation |
| (Narbute et al. 2019) | Parkinson’s disease/CNS (immunomodulation) | Differential ultracentrifugation |
| (Luo et al. 2021) | Bone loss (regeneration) | Differential ultracentrifugation |
| (Wu et al. 2021) | Regenerative endodontics | Differential ultracentrifugation |
| (Huang et al. 2024) | Tooth avulsion (regeneration) | Differential ultracentrifugation |
| (Li et al. 2022b) | Traumatic brain injury/CNS (anti-inflammation) | Precipitation |
| **PERIODONTAL LIGAMENT STEM CELLS-DERIVED EXOSOMES** | | |
| (Lei et al. 2022) | Periodontitis (regeneration) | Precipitation |
| (Huang et al. 2022a) | PDL/OTM (immunomodulation) | Differential ultracentrifugation & precipitation |
| (Diomede et al. 2018a) | PDL (regeneration) | Precipitation |
| (Zheng et al. 2023a) | PDL/OTM (immunomodulation) | Precipitation |
| (Lan et al. 2023) | Periodontitis (regeneration) | Differential ultracentrifugation |
| (Chiricosta et al. 2020) | Human carcinogenesis (regeneration) | Precipitation |
| (Niu et al. 2024) | Periodontitis (regeneration) | Differential ultracentrifugation |
| (Pizzicannella et al. 2019) | Bone defect (regeneration) | Precipitation |
| (Kang et al. 2018) | Periodontitis (immunomodulation) | Differential ultracentrifugation |
| (Wang et al. 2023a) | Bone defects (regeneration) | Differential ultracentrifugation & ultrafiltration |
| (Zhang et al. 2020b) | Periodontitis (regeneration) | Differential ultracentrifugation |
| (Zhao et al. 2022a) | Periodontitis (regeneration) | Differential ultracentrifugation |
| (Lu et al. 2023a) | Periodontitis (regeneration) | Differential ultracentrifugation |
| (Chang et al. 2023) | PDL/OTM (immunomodulation) | Differential ultracentrifugation |
| (Kang et al. 2023) | Periodontitis (anti-inflammation) | Differential ultracentrifugation |
| (Han et al. 2023a) | Bone defect (regeneration) | Differential ultracentrifugation & size-exclusion chromatography |
| (Zheng et al. 2019) | Periodontitis (immunomodulation) | Differential ultracentrifugation |
| (Wu et al. 2023) | Periodontitis (immunomodulation) | Ultrafiltration & precipitation |
| (Dai et al. 2022) | Periodontitis (regeneration) | Differential ultracentrifugation |
| (Novello et al. 2022) | Periodontitis (regeneration) | Precipitation |
| (Zhao et al. 2022b) | Periodontitis (regeneration) | Differential ultracentrifugation |
| (Cui et al. 2023) | Periodontitis (regeneration) | Differential ultracentrifugation |
| (Pourhajibagher and Bahador 2024) | Dental caries (antimicrobial) | Precipitation |
| (Lu et al. 2023b) | Periodontitis (regeneration) | Differential ultracentrifugation |
| (Soundara Rajan et al. 2017) | Multiple sclerosis (immunomodulation) | Precipitation |
| (Rajan et al. 2016) | Multiple sclerosis (regeneration) | Precipitation |
| (Xu et al. 2020) | Periodontitis (regeneration) | Precipitation |
| (Liu et al. 2020a) | Periodontitis (regeneration) | Differential ultracentrifugation |
| (Liu et al. 2023b) | Diabetes (immunomodulation) | Differential ultracentrifugation |
| (Han et al. 2024) | Craniofacial defect (regeneration) | Differential ultracentrifugation & size-exclusion chromatography |
| (Yu et al. 2023) | Periodontitis (regeneration) | Differential ultracentrifugation |
| (Xiang et al. 2024) | Periodontitis (regeneration) | Differential ultracentrifugation |
| (Giuliani et al. 2020) | Bone defect (regeneration) | Precipitation |
| **DENTAL PULP STEM CELLS-DERIVED EXOSOMES** | | |
| (Dong et al. 2021) | Salivary gland (immunomodulation) | Differential ultracentrifugation |
| (Chansaenroj et al. 2022) | Salivary gland (regeneration) | Differential ultracentrifugation |
| (Tian et al. 2023) | Bone defects (regeneration) | Differential ultracentrifugation |
| (Shen et al. 2020) | Periodontitis (regeneration) | Differential ultracentrifugation |
| (Zeng et al. 2022a) | Vital pulp therapy (regeneration) | Differential ultracentrifugation |
| (Li et al. 2023a) | Regeneration | Differential ultracentrifugation |
| (Vakhshiteh et al. 2021) | Carcinogenesis (regeneration) | Differential ultracentrifugation & precipitation |
| (Zhang et al. 2024) | Craniofacial defect | Differential ultracentrifugation |
| (Chen et al. 2021) | Dental pulp regeneration | Differential ultracentrifugation |
| (Han et al. 2023b) | Bone defect (regeneration) | Differential ultracentrifugation |
| (Huang et al. 2016) | Endodontics (regeneration) | Precipitation |
| (Guo et al. 2021a) | Tooth avulsion (regeneration) | Differential ultracentrifugation |
| (Swanson et al. 2020a) | Pulp capping (regeneration) | Differential ultracentrifugation |
| (Klimova et al. 2023) | Pancreatic cancer (anti-tumor) | Ultrafiltration & size-exclusive chromatography |
| (Ogata et al. 2024) | Sjogren’s syndrome (immunomodulation) | Differential ultracentrifugation |
| (Winkel et al. 2020) | Optimization | Differential ultracentrifugation |
| (Li et al. 2022d) | Dental pulp (regeneration) | Differential ultracentrifugation |
| (Swanson et al. 2020b) | Craniofacial defects (regeneration) | Differential ultracentrifugation & ultrafiltration |
| (Lin et al. 2024b) | Periodontitis (regeneration) | Ultrafiltration |
| (Mas-Bargues et al. 2023) | Oxidative stress (regeneration) | Differential ultracentrifugation |
| (Altanerova et al. 2016) | Intracerebral glioblastoma (antitumor) | Differential ultracentrifugation |
| (Nakatsuka et al. 2021) | Immunomodulation/regeneration | Precipitation |
| (Abdelgawad et al. 2022) | Dental pulp (regeneration) | Differential ultracentrifugation |
| (Venugopal et al. 2018) | Neurodegenerative disease/CNS (immunomodulation) | Differential ultracentrifugation |
| (Li et al. 2023b) | Optimization | Differential ultracentrifugation |
| (Fei et al. 2024) | Angiogenesis-related disorders (immunomodulation) | Differential ultracentrifugation |
| (Diomede et al. 2024) | Heart tissue regeneration | Precipitation |
| (Xie et al. 2020) | Bone defects (regeneration) | Differential ultracentrifugation |
| (Mas-Bargues et al. 2020) | Immunomodulation/regeneration | Differential ultracentrifugation |
| (Li et al. 2022a) | Regenerative endodontics | Differential ultracentrifugation |
| (Li et al. 2021b) | Cerebral ischemia (immunomodulation) | Differential ultracentrifugation & precipitation |
| (Zhou et al. 2021) | Regenerative endodontics | Differential ultracentrifugation |
| (Merckx et al. 2020) | Angiogenesis (regeneration) | Differential ultracentrifugation |
| (Chai et al. 2024a) | Sciatic nerve (regeneration) | Differential ultracentrifugation |
| (Wang et al. 2023d) | Dental pulp regeneration | Differential ultracentrifugation |
| (Li et al. 2021a) | Dental pulp (regeneration) | Differential ultracentrifugation |
| (Faruqu et al. 2020) | Optimization | Differential ultracentrifugation |
| (Sánchez-Sánchez et al. 2021) | Coronary artery disease (immunomodulation) | Differential ultracentrifugation |
| (Umar et al. 2024) | Immunomodulatory properties | Precipitation |
| (Guo et al. 2021b) | Mechanical stimulation | Differential ultracentrifugation |
| (Altanerova et al. 2017) | Hyperthermia (antitumor) | Size-exclusion chromatography |
| (Lin et al. 2021) | Osteoarthritis (regeneration) | Differential ultracentrifugation |
| (Zhou et al. 2022) | Cutaneous wound healing (regeneration) | Differential ultracentrifugation |
| (Zhang et al. 2020a) | Dental pulp (regeneration) | Differential ultracentrifugation |
| (Gómez-Ferrer et al. 2021) | MSCs immunomodulation | Differential ultracentrifugation |
| (Ganesh et al. 2022) | Dental pulp (regeneration) | Precipitation |
| (Fu et al. 2023b) | Osteoarthritis (regeneration) | Differential ultracentrifugation |
| (Teixeira et al. 2024) | Papillary thyroid cancer (antitumor) | Differential ultracentrifugation |
| (Chen et al. 2022) | Dental pulp (regeneration) | Differential ultracentrifugation & ultrafiltration |
| (Duan et al. 2024) | Optimization | Differential ultracentrifugation |
| (Ivica et al. 2020) | Regenerative endodontics | Differential ultracentrifugation |
| (Jin et al. 2020) | Craniofacial defect (regeneration) | Differential ultracentrifugation |
| (Terunuma et al. 2021) | Transcriptomics (regeneration) | Differential ultracentrifugation |
| (Lee et al. 2023) | Bone defect (regeneration) | Ultrafiltration & precipitation |
| (Eren Belgin et al. 2024) | Dental implant (anti-inflammation) | Precipitation |
| (Li and Ge 2022) | Dental pulp (immunomodulation) | Differential ultracentrifugation |
| (Nasiri 2023) | Regeneration dentistry | Differential ultracentrifugation & precipitation |
| (Brunello et al. 2022) | Craniofacial defects (regeneration) | Differential ultracentrifugation |
| (Kong et al. 2021) | Radiation injury (regeneration) | Differential ultracentrifugation |
| (Ji et al. 2019) | Immunological diseases (Immunomodulatory properties) | Differential ultracentrifugation |
| (Chai et al. 2024b) | Sciatic nerve injury (regeneration) | Differential ultracentrifugation |
| (Liu et al. 2022) | Spinal cord injury (immunomodulation) | Differential ultracentrifugation |
| (Liang et al. 2024b) | Subarachnoid hemorrhage (immunomodulation) | Differential ultracentrifugation |
| (Hu et al. 2023a) | Sjogren’s syndrome (antiinflammation & immunomodulation) | Differential ultracentrifugation |
| (Hu et al. 2019) | Dental pulp (immunomodulation) | Precipitation |
| (Zhou et al. 2020) | Regeneration | Differential ultracentrifugation |
| (Zheng et al. 2020) | Immunomodulation | Differential ultracentrifugation |
| (Luo et al. 2023) | Dental pulp (regeneration) | Differential ultracentrifugation |
| (Chi et al. 2024) | Immunomodulation (regeneration) | Differential ultracentrifugation |
| (Imanishi et al. 2021) | Bone defect (regeneration) | Differential ultracentrifugation |
| (Amaro-Prellezo et al. 2024) | Myocardial infarction (immunomodulation) | Ultrafiltration & size-exclusion chromatography |
| (Heitzer et al. 2023) | Bone defect (regeneration) | Differential ultracentrifugation |
| (Shimizu et al. 2022) | Periodontitis (regeneration) | Differential ultracentrifugation |
| (Miao et al. 2024) | Cerebral ischemia (regeneration/immunomodulation) | Differential ultracentrifugation |
| (Chen et al. 2024b) | Regeneration | Differential ultracentrifugation, ultrafiltration & size-exclusion chromatography |
| (Diomede et al. 2022) | Endodontic regeneration | Precipitation |
| **ALVEOLAR/JAWBONE STEM CELLS-DERIVED EXOSOMES** | | |
| (He et al. 2019) | Cutaneous wound healing | Differential ultracentrifugation & precipitation |
| (Li et al. 2020) | Bone defect (regeneration) | Differential ultracentrifugation |
| (Han et al. 2023a) | Optimization (bone regeneration) | Differential ultracentrifugation & size-exclusion chromatography |
| (Zhu et al. 2023) | Craniofacial defect (regeneration) | Differential ultracentrifugation |
| (Xu and Wang 2017) | Osteoporosis (regeneration) | Differential ultracentrifugation & precipitation |

**REFERENCES**

Abdelgawad LM, Nghnughi MH, Abdelgwad M. 2022. Influence of photo biomodulation using 980 nm diode laser and exsosomes derived from dental pulp stem cells on pulp regeneration of dogs’ teeth. reactions. 9:11.

Altanerova U, Babincova M, Babinec P, Benejova K, Jakubechova J, Altanerova V, Zduriencikova M, Repiska V, Altaner C. 2017. Human mesenchymal stem cell-derived iron oxide exosomes allow targeted ablation of tumor cells via magnetic hyperthermia. International Journal of Nanomedicine.7923-7936.

Altanerova U, Benejova K, Altanerova V, Tyciakova S, Rychly B, Szomolanyi P, Ciampor F, Cihova M, Repiska V, Ondicova K. 2016. Dental pulp mesenchymal stem/stromal cells labeled with iron sucrose release exosomes and cells applied intra-nasally migrate to intracerebral glioblastoma. Neoplasma. 63(6):925-933.

Amaro-Prellezo E, Gómez-Ferrer M, Hakobyan L, Ontoria-Oviedo I, Peiró-Molina E, Tarazona S, Salguero P, Ruiz-Saurí A, Selva-Roldán M, Vives-Sanchez R. 2024. Extracellular vesicles from dental pulp mesenchymal stem cells modulate macrophage phenotype during acute and chronic cardiac inflammation in athymic nude rats with myocardial infarction. Inflammation and Regeneration. 44(1):25.

Bastidas JG, Maurmann N, Scholl JN, Weber AF, Silveira RP, Figueiró F, Stimamiglio MA, Marcon B, Correa A, Pranke P. 2023. Secretome of stem cells from human exfoliated deciduous teeth (shed) and its extracellular vesicles improves keratinocytes migration, viability, and attenuation of h2o2‐induced cytotoxicity. Wound Repair and Regeneration. 31(6):827-841.

Bruckner S, Capria VM, Zeno B, Leblebicioglu B, Goyal K, Vasileff WK, Awan H, Willis WL, Ganesan LP, Jarjour WN. 2023. The therapeutic effects of gingival mesenchymal stem cells and their exosomes in a chimeric model of rheumatoid arthritis. Arthritis Research & Therapy. 25(1):211.

Brunello G, Zanotti F, Trentini M, Zanolla I, Pishavar E, Favero V, Favero R, Favero L, Bressan E, Bonora M. 2022. Exosomes derived from dental pulp stem cells show different angiogenic and osteogenic properties in relation to the age of the donor. Pharmaceutics. 14(5):908.

Chai Y, Liu Y, Liu Z, Wei W, Dong Y, Yang C, Chen M. 2024a. Dental pulp stem cell-derived exosomes promote sciatic nerve regeneration via optimizing schwann cell function. Cellular Reprogramming. 26(2):67-78.

Chai Y, Liu Y, Liu Z, Wei W, Dong Y, Yang C, Chen M. 2024b. Study on the role and mechanism of exosomes derived from dental pulp stem cells in promoting regeneration of myelin sheath in rats with sciatic nerve injury. Molecular Neurobiology.1-14.

Chang M, Chen Q, Wang B, Zhang Z, Han G. 2023. Exosomes from tension force-applied periodontal ligament cells promote mesenchymal stem cell recruitment by altering microrna profiles. International Journal of Stem Cells. 16(2):202-214.

Chansaenroj A, Adine C, Charoenlappanit S, Roytrakul S, Sariya L, Osathanon T, Rungarunlert S, Urkasemsin G, Chaisuparat R, Yodmuang S. 2022. Magnetic bioassembly platforms towards the generation of extracellular vesicles from human salivary gland functional organoids for epithelial repair. Bioactive Materials. 18:151-163.

Chen J, Shi X, Deng Y, Dang J, Liu Y, Zhao J, Liang R, Zeng D, Wu W, Xiong Y. 2024a. Mirna-148a–containing gmsc-derived evs modulate treg/th17 balance via ikkb/nf-κb pathway and treat a rheumatoid arthritis model. JCI insight. 9(10).

Chen T-Y, Huang T-Y, Chung Y-Y, Lin W-C, Lin H-Y, Chiu H-C, Lee S-Y. 2024b. Exosomes derived from polygonum multiflorum-treated human dental pulp stem cells (hdpscs): New approach in regenerative medicine. Journal of Drug Delivery Science and Technology. 99:105941.

Chen W-J, Xie J, Lin X, Ou M-H, Zhou J, Wei X-L, Chen W-X. 2021. The role of small extracellular vesicles derived from lipopolysaccharide-preconditioned human dental pulp stem cells in dental pulp regeneration. Journal of endodontics. 47(6):961-969.

Chen Y, Ma Y, Yang X, Chen J, Yang B, Tian W. 2022. The application of pulp tissue derived-exosomes in pulp regeneration: A novel cell-homing approach. International journal of nanomedicine.465-476.

Chi Y, Liu T, Jin Q, Liu H. 2024. Extracellular vesicles carrying runx3 promote differentiation of dental pulp stem cells. Tissue Engineering and Regenerative Medicine. 21(1):111-122.

Chiricosta L, Silvestro S, Gugliandolo A, Marconi GD, Pizzicannella J, Bramanti P, Trubiani O, Mazzon E. 2020. Extracellular vesicles of human periodontal ligament stem cells contain micrornas associated to proto-oncogenes: Implications in cytokinesis. Frontiers in genetics. 11:582.

Chu WX, Ding C, Du ZH, Wei P, Wang YX, Ge XJ, Yu GY. 2024. Shed‐exos promote saliva secretion by suppressing p‐erk1/2‐mediated apoptosis in glandular cells. Oral Diseases. 30(5):3066-3080.

Cui S, Zhang Z, Cheng C, Tang S, Zhai M, Li L, Wei F, Ding G. 2023. Small extracellular vesicles from periodontal ligament stem cells primed by lipopolysaccharide regulate macrophage m1 polarization via mir-433-3p targeting tlr2/tlr4/nf-κb. Inflammation. 46(5):1849-1858.

Dai Z, Li Z, Zheng W, Yan Z, Zhang L, Yang J, Xiao J, Sun H, Li S, Huang W. 2022. Gallic acid ameliorates the inflammatory state of periodontal ligament stem cells and promotes pro-osteodifferentiation capabilities of inflammatory stem cell-derived exosomes. Life. 12(9):1392.

Della Rocca Y, Diomede F, Konstantinidou F, Gatta V, Stuppia L, Benedetto U, Zimarino M, Lanuti P, Trubiani O, Pizzicannella J. 2024a. Autologous hgmsc-derived ips: A new proposal for tissue regeneration. International Journal of Molecular Sciences. 25(17):9169.

Della Rocca Y, Diomede F, Konstantinidou F, Trubiani O, Soundara Rajan T, Pierdomenico SD, Gatta V, Stuppia L, Marconi GD, Pizzicannella J. 2024b. Protective effect of oral stem cells extracellular vesicles on cardiomyocytes in hypoxia-reperfusion. Frontiers in Cell and Developmental Biology. 11:1260019.

Della Rocca Y, Pizzicannella J, Marco-ni GD, Fonticoli L, Trubiani O, Diomede F. 2022. Role of extra-cellular vesicles derived by human gingival mesenchymal stem cells in cardiomyocytes acute hypoxia. Italian Journal of Anatomy and Embryology. 126(2):35-38.

Deng Y, Liu Z, Lu M. 2024. Extracellular vesicles deviced from hypoxia-3d-gmscs rescue the mitochondrial dysfunction of aging-gmscs. Biochemical and Biophysical Research Communications. 717:150021.

Diomede F, D’aurora M, Gugliandolo A, Merciaro I, Ettorre V, Bramanti A, Piattelli A, Gatta V, Mazzon E, Fontana A. 2018a. A novel role in skeletal segment regeneration of extracellular vesicles released from periodontal-ligament stem cells. International journal of nanomedicine.3805-3825.

Diomede F, Fonticoli L, Marconi GD, Della Rocca Y, Rajan TS, Trubiani O, Murmura G, Pizzicannella J. 2022. Decellularized dental pulp, extracellular vesicles, and 5-azacytidine: A new tool for endodontic regeneration. Biomedicines. 10(2):403.

Diomede F, Guarnieri S, Lanuti P, Konstantinidou F, Gatta V, Rajan TS, Pierdomenico SD, Trubiani O, Marconi GD, Pizzicannella J. 2024. Extracellular vesicles (evs): A promising therapeutic tool in the heart tissue regeneration. BioFactors. 50(3):509-522.

Diomede F, Gugliandolo A, Cardelli P, Merciaro I, Ettorre V, Traini T, Bedini R, Scionti D, Bramanti A, Nanci A. 2018b. Three-dimensional printed pla scaffold and human gingival stem cell-derived extracellular vesicles: A new tool for bone defect repair. Stem Cell Research & Therapy. 9:1-21.

Dong J, Sakai K, Koma Y, Watanabe J, Liu K, Maruyama H, Sakaguchi K, Hibi H. 2021. Dental pulp stem cell-derived small extracellular vesicle in irradiation-induced senescence. Biochemical and Biophysical Research Communications. 575:28-35.

Du Z, Wei P, Jiang N, Wu L, Ding C, Yu G. 2023. Shed-derived exosomes ameliorate hyposalivation caused by sjögren’s syndrome via akt/gsk-3β/slug-mediated zo-1 expression. Chinese Medical Journal. 136(21):2596-2608.

Duan X, Zhang R, Feng H, Zhou H, Luo Y, Xiong W, Li J, He Y, Ye Q. 2024. A new subtype of artificial cell‐derived vesicles from dental pulp stem cells with the bioequivalence and higher acquisition efficiency compared to extracellular vesicles. Journal of Extracellular Vesicles. 13(7):e12473.

Eren Belgin E, Genç D, Tekin L, Sezgin S, Aladağ A. 2024. Anti‐inflammatory effect of dental pulpa mesenchymal stem cell exosomes loaded mucoadhesive hydrogel on mice with dental nickel hypersensitivity. Macromolecular Bioscience. 24(6):2300352.

Fallah A, Colagar AH, Khosravi A, Saeidi M. 2024. Exosomes from shed-msc regulate polarization and stress oxidative indexes in thp-1 derived m1 macrophages. Archives of Biochemistry and Biophysics. 755:109987.

Faruqu FN, Zhou S, Sami N, Gheidari F, Lu H, Al‐Jamal KT. 2020. Three‐dimensional culture of dental pulp pluripotent‐like stem cells (dppscs) enhances nanog expression and provides a serum‐free condition for exosome isolation. FASEB BioAdvances. 2(7):419.

Fei Y, Ling Z, Tong Q, Wang J. 2024. Apoptotic extracellular vesicles from supernumerary tooth-derived pulp stem cells transfer col1a1 to promote angiogenesis via pi3k/akt/vegf pathway. International Journal of Nanomedicine.6811-6828.

Fu H, Sen L, Zhang F, Liu S, Wang M, Mi H, Liu M, Li B, Peng S, Hu Z. 2023a. Mesenchymal stem cells-derived extracellular vesicles protect against oxidative stress-induced xenogeneic biological root injury via adaptive regulation of the pi3k/akt/nrf2 pathway. Journal of Nanobiotechnology. 21(1):466.

Fu Y, Cui S, Zhou Y, Qiu L. 2023b. Dental pulp stem cell-derived exosomes alleviate mice knee osteoarthritis by inhibiting trpv4-mediated osteoclast activation. International Journal of Molecular Sciences. 24(5):4926.

Ganesh V, Seol D, Gomez-Contreras PC, Keen HL, Shin K, Martin JA. 2022. Exosome-based cell homing and angiogenic differentiation for dental pulp regeneration. International journal of molecular sciences. 24(1):466.

Gao Y, Yuan Z, Yuan X, Wan Z, Yu Y, Zhan Q, Zhao Y, Han J, Huang J, Xiong C. 2022. Bioinspired porous microspheres for sustained hypoxic exosomes release and vascularized bone regeneration. Bioactive materials. 14:377-388.

Giuliani A, Sena G, Tromba G, Mazzon E, Fontana A, Diomede F, Piattelli A, Trubiani O. 2020. Could the enrichment of a biomaterial with conditioned medium or extracellular vesicles modify bone-remodeling kinetics during a defect healing? Evaluations on rat calvaria with synchrotron-based microtomography. Applied Sciences. 10(7):2336.

Gómez-Ferrer M, Villanueva-Badenas E, Sánchez-Sánchez R, Sánchez-López CM, Baquero MC, Sepúlveda P, Dorronsoro A. 2021. Hif-1α and pro-inflammatory signaling improves the immunomodulatory activity of msc-derived extracellular vesicles. International journal of molecular sciences. 22(7):3416.

Gratpain V, Loriot A, Bottemanne P, d’Auria L, Terrasi R, Payen VL, Van Pesch V, Muccioli GG, des Rieux A. 2024. Influence of a pro-inflammatory stimulus on the mirna and lipid content of human dental stem cell-derived extracellular vesicles and their impact on microglial activation. Heliyon. 10(5).

Guo H, Li B, Wu M, Zhao W, He X, Sui B, Dong Z, Wang L, Shi S, Huang X. 2021a. Odontogenesis-related developmental microenvironment facilitates deciduous dental pulp stem cell aggregates to revitalize an avulsed tooth. Biomaterials. 279:121223.

Guo J, Zhou F, Liu Z, Cao Y, Zhao W, Zhang Z, Zhai Q, Jin Y, Li B, Jin F. 2022. Exosome‐shuttled mitochondrial transcription factor a mrna promotes the osteogenesis of dental pulp stem cells through mitochondrial oxidative phosphorylation activation. Cell proliferation. 55(12):e13324.

Guo R, Fang Y, Zhang Y, Liu L, Li N, Wu J, Yan M, Li Z, Yu J. 2023. Shed-derived exosomes attenuate trigeminal neuralgia after cci of the infraorbital nerve in mice via the mir-24-3p/il-1r1/p-p38 mapk pathway. Journal of Nanobiotechnology. 21(1):458.

Guo S, Debbi L, Zohar B, Samuel R, Arzi RS, Fried AI, Carmon T, Shevach D, Redenski I, Schlachet I. 2021b. Stimulating extracellular vesicles production from engineered tissues by mechanical forces. Nano Letters. 21(6):2497-2504.

Hadady H, Karamali F, Ejeian F, Haghjooy Javanmard S, Rafiee L, Nasr Esfahani MH. 2021. Ac electrokinetic isolation and detection of extracellular vesicles from dental pulp stem cells: Theoretical simulation incorporating fluid mechanics. Electrophoresis. 42(20):2018-2026.

Hadady H, Karamali F, Ejeian F, Soroushzadeh S, Nasr-Esfahani MH. 2022. Potential neuroprotective effect of stem cells from apical papilla derived extracellular vesicles enriched by lab-on-chip approach during retinal degeneration. Cellular and Molecular Life Sciences. 79(7):350.

Han P, Johnson N, Abdal‐hay A, Moran CS, Salomon C, Ivanovski S. 2023a. Effects of periodontal cells‐derived extracellular vesicles on mesenchymal stromal cell function. Journal of Periodontal Research. 58(6):1188-1200.

Han P, Raveendran N, Liu C, Basu S, Jiao K, Johnson N, Moran CS, Ivanovski S. 2024. 3d bioprinted small extracellular vesicles from periodontal cells enhance mesenchymal stromal cell function. Biomaterials Advances. 158:213770.

Han S, Yang H, Ni X, Deng Y, Li Z, Xing X, Du M. 2023b. Programmed release of vascular endothelial growth factor and exosome from injectable chitosan nanofibrous microsphere-based plga-peg-plga hydrogel for enhanced bone regeneration. International Journal of Biological Macromolecules. 253:126721.

He X, Dong Z, Cao Y, Wang H, Liu S, Liao L, Jin Y, Yuan L, Li B. 2019. Msc‐derived exosome promotes m2 polarization and enhances cutaneous wound healing. Stem cells international. 2019(1):7132708.

Heitzer M, Zhao Q, Greven J, Winnand P, Zhang X, Bläsius FM, Buhl EM, Wolf M, Neuss S, Hildebrand F. 2023. Evaluation of in vitro biocompatibility of human pulp stem cells with allogeneic, alloplastic, and xenogeneic grafts under the influence of extracellular vesicles. Scientific Reports. 13(1):12475.

Hu S, Chen B, Zhou J, Liu F, Mao T, Pathak JL, Watanabe N, Li J. 2023a. Dental pulp stem cell-derived exosomes revitalize salivary gland epithelial cell function in nod mice via the gper-mediated camp/pka/creb signaling pathway. Journal of Translational Medicine. 21(1):361.

Hu X, Zhong Y, Kong Y, Chen Y, Feng J, Zheng J. 2019. Lineage-specific exosomes promote the odontogenic differentiation of human dental pulp stem cells (dpscs) through tgfβ1/smads signaling pathway via transfer of micrornas. Stem cell research & therapy. 10:1-14.

Hu Y, Wang Z, Fan C, Gao P, Wang W, Xie Y, Xu Q. 2023b. Human gingival mesenchymal stem cell‐derived exosomes cross‐regulate the wnt/β‐catenin and nf‐κb signalling pathways in the periodontal inflammation microenvironment. Journal of Clinical Periodontology. 50(6):796-806.

Huang C-C, Narayanan R, Alapati S, Ravindran S. 2016. Exosomes as biomimetic tools for stem cell differentiation: Applications in dental pulp tissue regeneration. Biomaterials. 111:103-115.

Huang H-m, Han C-S, Cui S-j, Zhou Y-k, Xin T-y, Zhang T, Zhu S-b, Zhou Y-h, Yang R-l. 2022a. Mechanical force-promoted osteoclastic differentiation via periodontal ligament stem cell exosomal protein anxa3. Stem Cell Reports. 17(8):1842-1858.

Huang T-Y, Chien M-S, Su W-T. 2022b. Therapeutic potential of pretreatment with exosomes derived from stem cells from the apical papilla against cisplatin-induced acute kidney injury. International Journal of Molecular Sciences. 23(10):5721.

Huang X, Sui B, Liu A, Guo H, Zheng C, Liu P, Cai X, Fu F, Bai S, Jin F. 2024. Odontogenesis‐empowered extracellular vesicles safeguard donor‐recipient stem cell interplay to support tooth regeneration. Small.2400260.

Huang Y, Liu Q, Liu L, Huo F, Guo S, Tian W. 2022c. Lipopolysaccharide-preconditioned dental follicle stem cells derived small extracellular vesicles treating periodontitis via reactive oxygen species/mitogen-activated protein kinase signaling-mediated antioxidant effect. International journal of nanomedicine.799-819.

Imanishi Y, Hata M, Matsukawa R, Aoyagi A, Omi M, Mizutani M, Naruse K, Ozawa S, Honda M, Matsubara T. 2021. Efficacy of extracellular vesicles from dental pulp stem cells for bone regeneration in rat calvarial bone defects. Inflammation and Regeneration. 41:1-10.

Ivica A, Ghayor C, Zehnder M, Valdec S, Weber FE. 2020. Pulp-derived exosomes in a fibrin-based regenerative root filling material. Journal of clinical medicine. 9(2):491.

Jarmalavičiūtė A, Tunaitis V, Pivoraitė U, Venalis A, Pivoriūnas A. 2015. Exosomes from dental pulp stem cells rescue human dopaminergic neurons from 6-hydroxy-dopamine–induced apoptosis. Cytotherapy. 17(7):932-939.

Ji L, Bao L, Gu Z, Zhou Q, Liang Y, Zheng Y, Xu Y, Zhang X, Feng X. 2019. Comparison of immunomodulatory properties of exosomes derived from bone marrow mesenchymal stem cells and dental pulp stem cells. Immunologic research. 67(4):432-442.

Jin Q, Li P, Yuan K, Zhao F, Zhu X, Zhang P, Huang Z. 2020. Extracellular vesicles derived from human dental pulp stem cells promote osteogenesis of adipose-derived stem cells via the mapk pathway. Journal of tissue engineering. 11:2041731420975569.

Jin S, Wang Y, Wu X, Li Z, Zhu L, Niu Y, Zhou Y, Liu Y. 2023. Young exosome bio‐nanoparticles restore aging‐impaired tendon stem/progenitor cell function and reparative capacity. Advanced Materials. 35(18):2211602.

Jing X, Wang S, Tang H, Li D, Zhou F, Xin L, He Q, Hu S, Zhang T, Chen T. 2022. Dynamically bioresponsive DNA hydrogel incorporated with dual-functional stem cells from apical papilla-derived exosomes promotes diabetic bone regeneration. ACS applied materials & interfaces. 14(14):16082-16099.

Jing Y, Zhao W, Zhou Z, Wang W, Niu Y, He X, Chang T, Guo C, Li B, Dou G. 2024. Apoptotic vesicles modulate endothelial metabolism and ameliorate ischemic retinopathy via pd1/pdl1 axis. Advanced Healthcare Materials.2303527.

Jonavičė U, Romenskaja D, Kriaučiūnaitė K, Jarmalavičiūtė A, Pajarskienė J, Kašėta V, Tunaitis V, Malm T, Giniatullin R, Pivoriūnas A. 2021. Extracellular vesicles from human teeth stem cells trigger atp release and promote migration of human microglia through p2x4 receptor/mfg-e8-dependent mechanisms. International Journal of Molecular Sciences. 22(20):10970.

Jonavičė U, Tunaitis V, Kriaučiūnaitė K, Jarmalavičiūtė A, Pivoriūnas A. 2019. Extracellular vesicles can act as a potent immunomodulators of human microglial cells. Journal of Tissue Engineering and Regenerative Medicine. 13(2):309-318.

Kang H, Lee M-J, Park SJ, Lee M-S. 2018. Lipopolysaccharide-preconditioned periodontal ligament stem cells induce m1 polarization of macrophages through extracellular vesicles. International journal of molecular sciences. 19(12):3843.

Kang L, Miao Y, Jin Y, Shen S, Lin X. 2023. Exosomal mir‐205‐5p derived from periodontal ligament stem cells attenuates the inflammation of chronic periodontitis via targeting xbp1. Immunity, Inflammation and Disease. 11(1):e743.

Katahira Y, Murakami F, Inoue S, Miyakawa S, Sakamoto E, Furusaka Y, Watanabe A, Sekine A, Kuroda M, Hasegawa H. 2023. Protective effects of conditioned media of immortalized stem cells from human exfoliated deciduous teeth on pressure ulcer formation. Frontiers in Immunology. 13:1010700.

Klimova D, Jakubechova J, Altanerova U, Nicodemou A, Styk J, Szemes T, Repiska V, Altaner C. 2023. Extracellular vesicles derived from dental mesenchymal stem/stromal cells with gemcitabine as a cargo have an inhibitory effect on the growth of pancreatic carcinoma cell lines in vitro. Molecular and Cellular Probes. 67:101894.

Koga S, Horiguchi Y. 2022. Efficacy of a cultured conditioned medium of exfoliated deciduous dental pulp stem cells in erectile dysfunction patients. Journal of cellular and molecular medicine. 26(1):195-201.

Kong F, Wu C-T, Geng P, Liu C, Xiao F, Wang L-S, Wang H. 2021. Dental pulp stem cell-derived extracellular vesicles mitigate haematopoietic damage after radiation. Stem Cell Reviews and Reports. 17(2):318-331.

Lan Q, Cao J, Bi X, Xiao X, Li D, Ai Y. 2023. Curcumin-primed periodontal ligament stem cells-derived extracellular vesicles improve osteogenic ability through the wnt/β-catenin pathway. Frontiers in Cell and Developmental Biology. 11:1225449.

Lee A, Choi J, Shi S, He P, Zhang Q, Le A. 2023. Dpsc-derived extracellular vesicles promote rat jawbone regeneration. Journal of Dental Research. 102(3):313-321.

Lei F, Li M, Lin T, Zhou H, Wang F, Su X. 2022. Treatment of inflammatory bone loss in periodontitis by stem cell-derived exosomes. Acta biomaterialia. 141:333-343.

Li B, Liang A, Zhou Y, Huang Y, Liao C, Zhang X, Gong Q. 2023a. Hypoxia preconditioned dpsc-derived exosomes regulate angiogenesis via transferring loxl2. Experimental cell research. 425(2):113543.

Li B, Xian X, Lin X, Huang L, Liang A, Jiang H, Gong Q. 2022a. Hypoxia alters the proteome profile and enhances the angiogenic potential of dental pulp stem cell-derived exosomes. Biomolecules. 12(4):575.

Li J, Ju Y, Liu S, Fu Y, Zhao S. 2021a. Exosomes derived from lipopolysaccharide-preconditioned human dental pulp stem cells regulate schwann cell migration and differentiation. Connective tissue research. 62(3):277-286.

Li L, Ge J. 2022. Exosome‑derived lncrna‑ankrd26 promotes dental pulp restoration by regulating mir‑150‑tlr4 signaling. Molecular medicine reports. 25(5):1-11.

Li M, Tian J, Yu K, Liu H, Yu X, Wang N, Gong Q, Li K, Shen Y, Wei X. 2024. A ros-responsive hydrogel incorporated with dental follicle stem cell-derived small extracellular vesicles promotes dental pulp repair by ameliorating oxidative stress. Bioactive Materials. 36:524-540.

Li S, Luo L, He Y, Li R, Xiang Y, Xing Z, Li Y, Albashari AA, Liao X, Zhang K. 2021b. Dental pulp stem cell‐derived exosomes alleviate cerebral ischaemia‐reperfusion injury through suppressing inflammatory response. Cell proliferation. 54(8):e13093.

Li X, Zheng Y, Hou L, Zhou Z, Huang Y, Zhang Y, Jia L, Li W. 2020. Exosomes derived from maxillary bmscs enhanced the osteogenesis in iliac bmscs. Oral Diseases. 26(1):131-144.

Li Y, Chen YH, Liu BY, Nie Q, Li LJ, Duan X, Wu LZ, Chen G. 2023b. Deciphering the heterogeneity landscape of mesenchymal stem/stromal cell‐derived extracellular vesicles for precise selection in translational medicine. Advanced healthcare materials. 12(15):2202453.

Li Y, Sun M, Wang X, Cao X, Li N, Pei D, Li A. 2022b. Dental stem cell-derived extracellular vesicles transfer mir-330-5p to treat traumatic brain injury by regulating microglia polarization. International journal of oral science. 14(1):44.

Li Y, Wang M, Sun M, Wang X, Pei D, Lei B, Li A. 2022c. Engineering antioxidant poly (citrate-gallic acid)-exosome hybrid hydrogel with microglia immunoregulation for traumatic brain injury-post neuro-restoration. Composites Part B: Engineering. 242:110034.

Li Y, Yang Y-Y, Ren J-L, Xu F, Chen F-M, Li A. 2017. Exosomes secreted by stem cells from human exfoliated deciduous teeth contribute to functional recovery after traumatic brain injury by shifting microglia m1/m2 polarization in rats. Stem Cell Research & Therapy. 8:1-11.

Li Z, Wu M, Liu S, Liu X, Huan Y, Ye Q, Yang X, Guo H, Liu A, Huang X. 2022d. Apoptotic vesicles activate autophagy in recipient cells to induce angiogenesis and dental pulp regeneration. Molecular Therapy. 30(10):3193-3208.

Liang L, Wang L, Liao Z, Ma L, Wang P, Zhao J, Wu J, Yang H. 2024a. High‐yield nanovesicles extruded from dental follicle stem cells promote the regeneration of periodontal tissues as an alternative of exosomes. Journal of Clinical Periodontology.

Liang X, Cai H, Sun H, Li N, Zhao W, Liu Q, Zeng Q, Wang Y, Gu Q. 2023. Nanoscale exosomes derived from gingiva mesenchymal stem cells for radiotherapy-induced apoptosis in non-small cell lung cancer cells. ACS Applied Nano Materials. 6(14):13533-13542.

Liang X, Miao Y, Tong X, Chen J, Liu H, He Z, Liu A, Hu Z. 2024b. Dental pulp mesenchymal stem cell-derived exosomes inhibit neuroinflammation and microglial pyroptosis in subarachnoid hemorrhage via the mirna-197-3p/foxo3 axis. Journal of Nanobiotechnology. 22(1):426.

Lin C-Y, Naruphontjirakul P, Huang T-Y, Wu Y-C, Cheng W-H, Su W-T. 2024a. The exosomes of stem cells from human exfoliated deciduous teeth suppress inflammation in osteoarthritis. International Journal of Molecular Sciences. 25(16):8560.

Lin T-Y, Huang T-Y, Chiu H-C, Chung Y-Y, Lin W-C, Lin H-Y, Lee S-Y. 2024b. 2, 3, 5, 4′-tetrahydroxystilbene-2-o-β-d-glucoside–stimulated dental pulp stem cells-derived exosomes for wound healing and bone regeneration. Journal of Dental Sciences.

Lin T, Wu N, Wang L, Zhang R, Pan R, Chen Y-F. 2021. Inhibition of chondrocyte apoptosis in a rat model of osteoarthritis by exosomes derived from mir‑140‑5p‑overexpressing human dental pulp stem cells. International Journal of Molecular Medicine. 47(3):1-1.

Lin X, Wang H, Wu T, Zhu Y, Jiang L. 2023. Exosomes derived from stem cells from apical papilla promote angiogenesis via mir‐126 under hypoxia. Oral diseases. 29(8):3408-3419.

Liu C, Hu F, Jiao G, Guo Y, Zhou P, Zhang Y, Zhang Z, Yi J, You Y, Li Z. 2022. Dental pulp stem cell-derived exosomes suppress m1 macrophage polarization through the ros-mapk-nfκb p65 signaling pathway after spinal cord injury. Journal of nanobiotechnology. 20(1):65.

Liu D, Shi B, Zhou W, Tao G. 2023a. Exosomes from hypoxia-conditioned apical papilla stem cells accelerate angiogenesis in vitro through notch/jag1/vegf signaling. Tissue and Cell. 84:102197.

Liu F, Wang X, Xu J, Lu Y, Bai Y, Lv J. 2024. Preliminary study on the mechanism by which exosomes derived from human exfoliated deciduous teeth improve the proliferation and osteogenic inhibitory effect of glucocorticoid-induced bmscs. Gene. 923:148575.

Liu M, Chen R, Xu Y, Zheng J, Wang M, Wang P. 2023b. Exosomal mir‐141‐3p from pdlscs alleviates high glucose‐induced senescence of pdlscs by activating the keap1‐nrf2 signaling pathway. Stem Cells International. 2023(1):7136819.

Liu T, Hu W, Zou X, Xu J, He S, Chang L, Li X, Yin Y, Tian M, Li Z. 2020a. Human periodontal ligament stem cell‐derived exosomes promote bone regeneration by altering microrna profiles. Stem Cells International. 2020(1):8852307.

Liu Y, Zhuang X, Yu S, Yang N, Zeng J, Chen X. 2020b. Exosomes derived from stem cells from apical papilla promote craniofacial soft tissue regeneration through enhancing cdc42-mediated vascularization.

Liu Z, Yang S, Li X, Wang S, Zhang T, Huo N, Duan R, Shi Q, Zhang J, Xu J. 2023c. Local transplantation of gmsc-derived exosomes to promote vascularized diabetic wound healing by regulating the wnt/β-catenin pathways. Nanoscale Advances. 5(3):916-926.

Lu H, Mu Q, Ku W, Zheng Y, Yi P, Lin L, Li P, Wang B, Wu J, Yu D. 2024. Functional extracellular vesicles from sheds combined with gelatin methacryloyl promote the odontogenic differentiation of dpscs for pulp regeneration. Journal of Nanobiotechnology. 22(1):265.

Lu J, Yu N, Liu Q, Xie Y, Zhen L. 2023a. Periodontal ligament stem cell exosomes key to regulate periodontal regeneration by mir-31-5p in mice model. International Journal of Nanomedicine.5327-5342.

Lu Y, Zhao L, Mao J, Liu W, Ma W, Zhao B. 2023b. Rab27a-mediated extracellular vesicle secretion contributes to osteogenesis in periodontal ligament-bone niche communication. Scientific Reports. 13(1):8479.

Luo L, Avery SJ, Waddington RJ. 2021. Exploring a chemotactic role for evs from progenitor cell populations of human exfoliated deciduous teeth for promoting migration of naïve bmscs in bone repair process. Stem cells international. 2021(1):6681771.

Luo P, Jiang C, Ji P, Wang M, Xu J. 2019. Exosomes of stem cells from human exfoliated deciduous teeth as an anti-inflammatory agent in temporomandibular joint chondrocytes via mir-100-5p/mtor. Stem Cell Research & Therapy. 10:1-12.

Luo X, Feng W, Huang S, Miao S, Jiang T, Lei Q, Yin J, Zhang S, Bai X, Hao C. 2023. Odontoblasts release exosomes to regulate the odontoblastic differentiation of dental pulp stem cells. Stem Cell Research & Therapy. 14(1):176.

Ma L, Rao N, Jiang H, Dai Y, Yang S, Yang H, Hu J. 2022. Small extracellular vesicles from dental follicle stem cells provide biochemical cues for periodontal tissue regeneration. Stem Cell Research & Therapy. 13(1):92.

Mao E, Hu Y, Xin Y, Sun Z, Zhang J, Li S. 2023. Human dental follicle cell‐derived small extracellular vesicles attenuate temporomandibular joint cartilage damage through inhibiting hif‐2α. Journal of Tissue Engineering and Regenerative Medicine. 2023(1):6625123.

Mas-Bargues C, Sanz-Ros J, Román-Domínguez A, Gimeno-Mallench L, Inglés M, Viña J, Borrás C. 2020. Extracellular vesicles from healthy cells improves cell function and stemness in premature senescent stem cells by mir-302b and hif-1α activation. Biomolecules. 10(6):957.

Mas-Bargues C, Sanz-Ros J, Romero-García N, Huete-Acevedo J, Dromant M, Borrás C. 2023. Small extracellular vesicles from senescent stem cells trigger adaptive mechanisms in young stem cells by increasing antioxidant enzyme expression. Redox Biology. 62:102668.

Merckx G, Hosseinkhani B, Kuypers S, Deville S, Irobi J, Nelissen I, Michiels L, Lambrichts I, Bronckaers A. 2020. Angiogenic effects of human dental pulp and bone marrow-derived mesenchymal stromal cells and their extracellular vesicles. Cells. 9(2):312.

Miao Y, Liang X, Chen J, Liu H, He Z, Qin Y, Liu A, Zhang R. 2024. Transfer of mir-877–3p via extracellular vesicles derived from dental pulp stem cells attenuates neuronal apoptosis and facilitates early neurological functional recovery after cerebral ischemia–reperfusion injury through the bclaf1/p53 signaling pathway. Pharmacological Research. 206:107266.

Nakatsuka R, Sasaki Y, Masutani M, Nozaki T. 2021. Parp1 regulates cellular processes mediated by exosomal mirnas in dental pulp stem cells. Journal of Hard Tissue Biology. 30(4):371-378.

Narbute K, Piļipenko V, Pupure J, Dzirkale Z, Jonavičė U, Tunaitis V, Kriaučiūnaitė K, Jarmalavičiūtė A, Jansone B, Kluša V. 2019. Intranasal administration of extracellular vesicles derived from human teeth stem cells improves motor symptoms and normalizes tyrosine hydroxylase expression in the substantia nigra and striatum of the 6-hydroxydopamine-treated rats. Stem Cells Translational Medicine. 8(5):490-499.

Nasiri K. 2023. Exosomes derived from human dental stem cell enhance the viability of odontoblasts. Nanomedicine Research Journal. 8(4):365-372.

Nie Y-F, Shang J-M, Liu D-Q, Meng W-Q, Ren H-P, Li C-H, Wang Z-F, Lan J. 2024. Apical papilla stem cell-derived exosomes regulate lipid metabolism and alleviate inflammation in the mcd-induced mouse nash model. Biochemical Pharmacology. 222:116073.

Niu Q, Lin C, Yang S, Rong S, Wei J, Zhao T, Peng Y, Cheng Z, Xie Y, Wang Y. 2024. Foxo1-overexpressed small extracellular vesicles derived from hpdlscs promote periodontal tissue regeneration by reducing mitochondrial dysfunction to regulate osteogenesis and inflammation. International Journal of Nanomedicine.8751-8768.

Novello S, Tricot-Doleux S, Novella A, Pellen-Mussi P, Jeanne S. 2022. Influence of periodontal ligament stem cell-derived conditioned medium on osteoblasts. Pharmaceutics. 14(4):729.

Ogata K, Moriyama M, Kawado T, Yoshioka H, Yano A, Matsumura-Kawashima M, Nakamura S, Kawano S. 2024. Extracellular vesicles of ips cells highly capable of producing hgf and tgf-β1 can attenuate sjögren's syndrome via innate immunity regulation. Cellular Signalling. 113:110980.

Peng Y, Zhao T, Rong S, Yang S, Teng W, Xie Y, Wang Y. 2024. Young small extracellular vesicles rejuvenate replicative senescence by remodeling drp1 translocation-mediated mitochondrial dynamics. Journal of Nanobiotechnology. 22(1):543.

Pivoraitė U, Jarmalavičiūtė A, Tunaitis V, Ramanauskaitė G, Vaitkuvienė A, Kašėta V, Biziulevičienė G, Venalis A, Pivoriūnas A. 2015. Exosomes from human dental pulp stem cells suppress carrageenan-induced acute inflammation in mice. Inflammation. 38:1933-1941.

Pizzicannella J, Gugliandolo A, Orsini T, Fontana A, Ventrella A, Mazzon E, Bramanti P, Diomede F, Trubiani O. 2019. Engineered extracellular vesicles from human periodontal-ligament stem cells increase vegf/vegfr2 expression during bone regeneration. Frontiers in physiology. 10:512.

Pourhajibagher M, Bahador A. 2024. Periodontal ligament stem cell-derived exosome-loaded emodin mediated antimicrobial photodynamic therapy against cariogenic bacteria. BMC Oral Health. 24(1):311.

Rajan TS, Giacoppo S, Diomede F, Ballerini P, Paolantonio M, Marchisio M, Piattelli A, Bramanti P, Mazzon E, Trubiani O. 2016. The secretome of periodontal ligament stem cells from ms patients protects against eae. Scientific Reports. 6(1):38743.

Rao F, Zhang D, Fang T, Lu C, Wang B, Ding X, Wei S, Zhang Y, Pi W, Xu H. 2019. Exosomes from human gingiva‐derived mesenchymal stem cells combined with biodegradable chitin conduits promote rat sciatic nerve regeneration. Stem cells international. 2019(1):2546367.

Sánchez-Sánchez R, Gómez-Ferrer M, Reinal I, Buigues M, Villanueva-Bádenas E, Ontoria-Oviedo I, Hernándiz A, González-King H, Peiró-Molina E, Dorronsoro A. 2021. Mir-4732-3p in extracellular vesicles from mesenchymal stromal cells is cardioprotective during myocardial ischemia. Frontiers in cell and developmental biology. 9:734143.

Shen Z, Kuang S, Zhang Y, Yang M, Qin W, Shi X, Lin Z. 2020. Chitosan hydrogel incorporated with dental pulp stem cell-derived exosomes alleviates periodontitis in mice via a macrophage-dependent mechanism. Bioactive materials. 5(4):1113-1126.

Shi Q, Qian Z, Liu D, Sun J, Wang X, Liu H, Xu J, Guo X. 2017. Gmsc-derived exosomes combined with a chitosan/silk hydrogel sponge accelerates wound healing in a diabetic rat skin defect model. Frontiers in physiology. 8:904.

Shimizu Y, Takeda‐Kawaguchi T, Kuroda I, Hotta Y, Kawasaki H, Hariyama T, Shibata T, Akao Y, Kunisada T, Tatsumi J. 2022. Exosomes from dental pulp cells attenuate bone loss in mouse experimental periodontitis. Journal of periodontal research. 57(1):162-172.

Silvestro S, Chiricosta L, Gugliandolo A, Pizzicannella J, Diomede F, Bramanti P, Trubiani O, Mazzon E. 2020. Extracellular vesicles derived from human gingival mesenchymal stem cells: A transcriptomic analysis. Genes. 11(2):118.

Sonoda S, Murata S, Kato H, Zakaria F, Kyumoto-Nakamura Y, Uehara N, Yamaza H, Kukita T, Yamaza T. 2021. Targeting of deciduous tooth pulp stem cell–derived extracellular vesicles on telomerase-mediated stem cell niche and immune regulation in systemic lupus erythematosus. The Journal of Immunology. 206(12):3053-3063.

Sonoda S, Murata S, Nishida K, Kato H, Uehara N, Kyumoto YN, Yamaza H, Takahashi I, Kukita T, Yamaza T. 2020. Extracellular vesicles from deciduous pulp stem cells recover bone loss by regulating telomerase activity in an osteoporosis mouse model. Stem Cell Research & Therapy. 11:1-16.

Soundara Rajan T, Giacoppo S, Diomede F, Bramanti P, Trubiani O, Mazzon E. 2017. Human periodontal ligament stem cells secretome from multiple sclerosis patients suppresses nalp3 inflammasome activation in experimental autoimmune encephalomyelitis. International journal of immunopathology and pharmacology. 30(3):238-252.

Sun J, Wang Z, Liu P, Hu Y, Li T, Yang J, Gao P, Xu Q. 2022. Exosomes derived from human gingival mesenchymal stem cells attenuate the inflammatory response in periodontal ligament stem cells. Frontiers in Chemistry. 10:863364.

Sunartvanichkul T, Arayapisit T, Sangkhamanee SS, Chaweewannakorn C, Iwasaki K, Klaihmon P, Sritanaudomchai H. 2023. Stem cell-derived exosomes from human exfoliated deciduous teeth promote angiogenesis in hyperglycemic-induced human umbilical vein endothelial cells. Journal of Applied Oral Science. 31:e20220427.

Swanson WB, Gong T, Zhang Z, Eberle M, Niemann D, Dong R, Rambhia KJ, Ma PX. 2020a. Controlled release of odontogenic exosomes from a biodegradable vehicle mediates dentinogenesis as a novel biomimetic pulp capping therapy. Journal of Controlled Release. 324:679-694.

Swanson WB, Zhang Z, Xiu K, Gong T, Eberle M, Wang Z, Ma PX. 2020b. Scaffolds with controlled release of pro-mineralization exosomes to promote craniofacial bone healing without cell transplantation. Acta Biomaterialia. 118:215-232.

Teixeira MR, Alievi AL, da Costa VR, Kerkis I, Araldi RP. 2024. Exploring the therapeutic potential of extracellular vesicles derived from human immature dental pulp cells on papillary thyroid cancer. International Journal of Molecular Sciences. 25(15).

Terunuma A, Yoshioka Y, Sekine T, Takane T, Shimizu Y, Narita S, Ochiya T, Terunuma H. 2021. Extracellular vesicles from mesenchymal stem cells of dental pulp and adipose tissue display distinct transcriptomic characteristics suggestive of potential therapeutic targets. Journal of Stem Cells & Regenerative Medicine. 17(2):56.

Tian J, Chen W, Xiong Y, Li Q, Kong S, Li M, Pang C, Qiu Y, Xu Z, Gong Q. 2023. Small extracellular vesicles derived from hypoxic preconditioned dental pulp stem cells ameliorate inflammatory osteolysis by modulating macrophage polarization and osteoclastogenesis. Bioactive Materials. 22:326-342.

Tian X, Wei W, Cao Y, Ao T, Huang F, Javed R, Wang X, Fan J, Zhang Y, Liu Y. 2022. Gingival mesenchymal stem cell‐derived exosomes are immunosuppressive in preventing collagen‐induced arthritis. Journal of Cellular and Molecular Medicine. 26(3):693-708.

Umar S, Debnath K, Leung K, Huang C-C, Lu Y, Gajendrareddy P, Ravindran S. 2024. Immunomodulatory properties of naïve and inflammation-informed dental pulp stem cell derived extracellular vesicles. Frontiers in Immunology. 15:1447536.

Vakhshiteh F, Rahmani S, Ostad SN, Madjd Z, Dinarvand R, Atyabi F. 2021. Exosomes derived from mir-34a-overexpressing mesenchymal stem cells inhibit in vitro tumor growth: A new approach for drug delivery. Life Sciences. 266:118871.

Venugopal C, K S, Rai KS, Pinnelli VB, Kutty BM, Dhanushkodi A. 2018. Neuroprotection by human dental pulp mesenchymal stem cells: From billions to nano. Current gene therapy. 18(5):307-323.

Wang A, Liu J, Zhuang X, Yu S, Zhu S, Liu Y, Chen X. 2020a. Identification and comparison of pirna expression profiles of exosomes derived from human stem cells from the apical papilla and bone marrow mesenchymal stem cells. Stem Cells and Development. 29(8):511-520.

Wang H, Yang F, Wang Y, Pei F, Chen Z, Zhang L. 2019. Odontoblastic exosomes attenuate apoptosis in neighboring cells. Journal of dental research. 98(11):1271-1278.

Wang J, Qiao Q, Sun Y, Yu W, Wang J, Zhu M, Yang K, Huang X, Bai Y. 2023a. Osteogenic differentiation effect of human periodontal ligament stem-cell initial cell density on autologous cells and human bone marrow stromal cells. International Journal of Molecular Sciences. 24(8):7133.

Wang M, Li J, Ye Y, Chen D, Song J. 2023b. Shed‐derived exosomes improve the repair capacity and osteogenesis potential of hpdlcs. Oral Diseases. 29(4):1692-1705.

Wang M, Li J, Ye Y, He S, Song J. 2020b. Shed-derived conditioned exosomes enhance the osteogenic differentiation of pdlscs via wnt and bmp signaling in vitro. Differentiation. 111:1-11.

Wang R, Ji Q, Meng C, Liu H, Fan C, Lipkind S, Wang Z, Xu Q. 2020c. Role of gingival mesenchymal stem cell exosomes in macrophage polarization under inflammatory conditions. International immunopharmacology. 81:106030.

Wang S, Liu Z, Yang S, Huo N, Qiao B, Zhang T, Xu J, Shi Q. 2023c. Extracellular vesicles secreted by human gingival mesenchymal stem cells promote bone regeneration in rat femoral bone defects. Frontiers in Bioengineering and Biotechnology. 11:1098172.

Wang S, Xing X, Peng W, Huang C, Du Y, Yang H, Zhou J. 2023d. Fabrication of an exosome-loaded thermosensitive chitin-based hydrogel for dental pulp regeneration. Journal of Materials Chemistry B. 11(7):1580-1590.

Wei J, Song Y, Du Z, Yu F, Zhang Y, Jiang N, Ge X. 2020. Exosomes derived from human exfoliated deciduous teeth ameliorate adult bone loss in mice through promoting osteogenesis. Journal of molecular histology. 51:455-466.

Winkel A, Jaimes Y, Melzer C, Dillschneider P, Hartwig H, Stiesch M, von der Ohe J, Strauss S, Vogt PM, Hamm A. 2020. Cell culture media notably influence properties of human mesenchymal stroma/stem-like cells from different tissues. Cytotherapy. 22(11):653-668.

Wu J, Chen L, Wang R, Song Z, Shen Z, Zhao Y, Huang S, Lin Z. 2019. Exosomes secreted by stem cells from human exfoliated deciduous teeth promote alveolar bone defect repair through the regulation of angiogenesis and osteogenesis. ACS biomaterials science & engineering. 5(7):3561-3571.

Wu M, Liu X, Li Z, Huang X, Guo H, Guo X, Yang X, Li B, Xuan K, Jin Y. 2021. Shed aggregate exosomes shuttled mir‐26a promote angiogenesis in pulp regeneration via tgf‐β/smad2/3 signalling. Cell proliferation. 54(7):e13074.

Wu Y, Qu F, Zhang Y, Song Y, Zhong Q, Huang Y, Wang Y, Cao X, Fan Z, Xu C. 2023. Exosomes from cyclic stretched periodontal ligament cells induced periodontal inflammation through mir-9-5p/sirt1/nf-κb signaling pathway. The Journal of Immunology. 210(12):2001-2015.

Xiang M, Liu Y, Guo Q, Liao C, Xiao L, Xiang M, Guan X, Liu J. 2024. Metformin enhances the therapeutic effects of extracellular vesicles derived from human periodontal ligament stem cells on periodontitis. Scientific Reports. 14(1):19940.

Xie L, Guan Z, Zhang M, Lyu S, Thuaksuban N, Kamolmattayakul S, Nuntanaranont T. 2020. Exosomal circlpar1 promoted osteogenic differentiation of homotypic dental pulp stem cells by competitively binding to hsa‐mir‐31. Biomed Research International. 2020(1):6319395.

Xie Y, Yu L, Cheng Z, Peng Y, Cao Z, Chen B, Duan Y, Wang Y. 2022. Shed-derived exosomes promote lps-induced wound healing with less itching by stimulating macrophage autophagy. Journal of Nanobiotechnology. 20(1):239.

Xu S, Wang Z. 2017. Bone marrow mesenchymal stem cell-derived exosomes enhance osteoclastogenesis during alveolar bone deterioration in rats. RSC advances. 7(34):21153-21163.

Xu X-Y, Tian B-M, Xia Y, Xia Y-L, Li X, Zhou H, Tan Y-Z, Chen F-M. 2020. Exosomes derived from p2x7 receptor gene-modified cells rescue inflammation-compromised periodontal ligament stem cells from dysfunction. Stem cells translational medicine. 9(11):1414-1430.

Yang S, Liu Q, Chen S, Zhang F, Li Y, Fan W, Mai L, He H, Huang F. 2022. Extracellular vesicles delivering nuclear factor i/c for hard tissue engineering: Treatment of apical periodontitis and dentin regeneration. Journal of Tissue Engineering. 13:20417314221084095.

Yi G, Zhang S, Ma Y, Yang X, Huo F, Chen Y, Yang B, Tian W. 2022. Matrix vesicles from dental follicle cells improve alveolar bone regeneration via activation of the plc/pkc/mapk pathway. Stem Cell Research & Therapy. 13(1):41.

Yu J, Wu X, Zhang W, Chu F, Zhang Q, Gao M, Xu Y, Wu Y. 2023. Effect of psoralen on the regulation of osteogenic differentiation induced by periodontal stem cell-derived exosomes. Human Cell. 36(4):1389-1402.

Yu S, Chen X, Liu Y, Zhuang XY, Wang AC, Liu XM, Zhu S. 2022a. Exosomes derived from stem cells from the apical papilla alleviate inflammation in rat pulpitis by upregulating regulatory t cells. International Endodontic Journal. 55(5):517-530.

Yu T, Mi N, Song Y, Xie W. 2024. Exosomes mir‐92a‐3p from human exfoliated deciduous teeth inhibits periodontitis progression via the klf4/pi3k/akt pathway. Journal of Periodontal Research.

Yu Z, Wen Y, Jiang N, Li Z, Guan J, Zhang Y, Deng C, Zhao L, Zheng SG, Zhu Y. 2022b. Tnf-α stimulation enhances the neuroprotective effects of gingival mscs derived exosomes in retinal ischemia-reperfusion injury via the meg3/mir-21a-5p axis. Biomaterials. 284:121484.

Zarubova J, Hasani‐Sadrabadi MM, Dashtimoghadam E, Zhang X, Ansari S, Li S, Moshaverinia A. 2022. Engineered delivery of dental stem‐cell‐derived extracellular vesicles for periodontal tissue regeneration. Advanced healthcare materials. 11(12):2102593.

Zeng J, He K, Mai R, Lin T, Wei R, Nong J, Wu Y. 2022a. Exosomes from human umbilical cord mesenchymal stem cells and human dental pulp stem cells ameliorate lipopolysaccharide-induced inflammation in human dental pulp stem cells. Archives of Oral Biology. 138:105411.

Zeng T, Yuan P, Liang L, Zhang X, Zhang H, Wu W. 2022b. Cartilaginous extracellular matrix enriched with human gingival mesenchymal stem cells derived “matrix bound extracellular vesicles” enabled functional reconstruction of tracheal defect. Advanced Science. 9(2):2102735.

Zhang S, Thiebes AL, Kreimendahl F, Ruetten S, Buhl EM, Wolf M, Jockenhoevel S, Apel C. 2020a. Extracellular vesicles-loaded fibrin gel supports rapid neovascularization for dental pulp regeneration. International Journal of Molecular Sciences. 21(12):4226.

Zhang S, Wang S, Chen J, Cui Y, Lu X, Xiong S, Yue C, Yang B. 2024. Human dental pulp stem cell-derived exosomes decorated titanium scaffolds for promoting bone regeneration. Colloids and Surfaces B: Biointerfaces. 235:113775.

Zhang T, Chen Z, Zhu M, Jing X, Xu X, Yuan X, Zhou M, Zhang Y, Lu M, Chen D. 2023. Extracellular vesicles derived from human dental mesenchymal stem cells stimulated with low-intensity pulsed ultrasound alleviate inflammation-induced bone loss in a mouse model of periodontitis. Genes & Diseases. 10(4):1613-1625.

Zhang Y, Shi S, Xu Q, Zhang Q, Shanti R, Le A. 2019. Sis-ecm laden with gmsc-derived exosomes promote taste bud regeneration. Journal of dental research. 98(2):225-233.

Zhang Y, Wang Z, Shi B, Li Y, Wang R, Sun J, Hu Y, Yuan C, Xu Q. 2021. Effect of gingival mesenchymal stem cell-derived exosomes on inflammatory macrophages in a high-lipid microenvironment. International immunopharmacology. 94:107455.

Zhang Z, Shuai Y, Zhou F, Yin J, Hu J, Guo S, Wang Y, Liu W. 2020b. Pdlscs regulate angiogenesis of periodontal ligaments via vegf transferred by exosomes in periodontitis. International journal of medical sciences. 17(5):558.

Zhao B, Chen Q, Zhao L, Mao J, Huang W, Han X, Liu Y. 2022a. Periodontal ligament stem cell-derived small extracellular vesicles embedded in matrigel enhance bone repair through the adenosine receptor signaling pathway. International Journal of Nanomedicine.519-536.

Zhao Y, Gong Y, Liu X, He J, Zheng B, Liu Y. 2022b. The experimental study of periodontal ligament stem cells derived exosomes with hydrogel accelerating bone regeneration on alveolar bone defect. Pharmaceutics. 14(10):2189.

Zheng J, Kong Y, Hu X, Li Z, Li Y, Zhong Y, Wei X, Ling J. 2020. Microrna-enriched small extracellular vesicles possess odonto-immunomodulatory properties for modulating the immune response of macrophages and promoting odontogenesis. Stem cell research & therapy. 11:1-14.

Zheng X, Zhao N, Peng L, Li Z, Liu C, You Q, Fang B. 2023a. Biological characteristics of micrornas secreted by exosomes of periodontal ligament stem cells due to mechanical force. European Journal of Orthodontics. 45(4):408-417.

Zheng Y, Dong C, Yang J, Jin Y, Zheng W, Zhou Q, Liang Y, Bao L, Feng G, Ji J. 2019. Exosomal microrna‐155‐5p from pdlscs regulated th17/treg balance by targeting sirtuin‐1 in chronic periodontitis. Journal of Cellular Physiology. 234(11):20662-20674.

Zheng Y, Lu H, Mu Q, Yi P, Lin L, Li P, Yu D, Zhao W. 2023b. Effects of sev derived from shed and dpsc on the proliferation, migration and osteogenesis of pdlsc. Regenerative Therapy. 24:489-498.

Zhou H, Li X, Wu RX, He XT, An Y, Xu XY, Sun HH, Wu LA, Chen FM. 2021. Periodontitis‐compromised dental pulp stem cells secrete extracellular vesicles carrying mirna‐378a promote local angiogenesis by targeting sufu to activate the hedgehog/gli1 signalling. Cell proliferation. 54(5):e13026.

Zhou H, Li X, Yin Y, He X-T, An Y, Tian B-M, Hong Y-L, Wu L-A, Chen F-M. 2020. The proangiogenic effects of extracellular vesicles secreted by dental pulp stem cells derived from periodontally compromised teeth. Stem cell research & therapy. 11:1-18.

Zhou Z, Zheng J, Lin D, Xu R, Chen Y, Hu X. 2022. Exosomes derived from dental pulp stem cells accelerate cutaneous wound healing by enhancing angiogenesis via the cdc42/p38 mapk pathway. International journal of molecular medicine. 50(6):1-15.

Zhu Q, Tang Y, Zhou T, Yang L, Zhang G, Meng Y, Zhang H, Gao J, Wang C, Su Y-X. 2023. Exosomes derived from mesenchymal stromal cells promote bone regeneration by delivering mir-182–5p-inhibitor. Pharmacological research. 192:106798.

Zhuang X, Ji L, Jiang H, Liu Y, Liu X, Bi J, Zhao W, Ding Z, Chen X. 2020. Exosomes derived from stem cells from the apical papilla promote dentine‐pulp complex regeneration by inducing specific dentinogenesis. Stem cells international. 2020(1):5816723.
