# Supplementary material for: Isolation methods of exosomes derived from dental stem cells
Source: Int J Oral Sci. 2025 Jun 16;17:50. doi: 10.1038/s41368-025-00370-y (PMC12170887; doi:10.1038/s41368-025-00370-y)
Supplement: Supplementary file 3 — Supplemental Table S3 [file 41368_2025_370_MOESM3_ESM.docx]

**Table S3**. Comparison of the most widely used exosome isolation method (differential ultracentrifugation) with label-free exosome isolation methods.

| **Isolation method** | **Specimen** | **Sample volume** | **Time required** | **Size range** | **Recovery rate** | **Purity** | **Reference** |
| --- | --- | --- | --- | --- | --- | --- | --- |
| **Most widely used approach** | | | | | | | |
| Differential ultracentrifugation | Serum, plasma, cell culture supernatant | >10 mL | ≥4 h | NA | 5-25% | 187 ng RNA/100 ug protein (from 5 mL sample) | Li et al. 2017 (101) |
| **Sieving separation** | | | | | | | |
| - Single tangential filtration - Zinc oxide nanowire microfluidic device - Ciliated micropillar array - ExoTIC (multiple filtration) - Exodisc (centrifugal+double filtration) - Double-dead end filtration | - Plasma & Cell culture supernatant - Urine - Liposomes suspended in PBS - Plasma, urine, & cell culture supernatant - Urine & cell culture supernatant - Urine & cell culture supernatant | - 500 uL - 1 mL - 30-100 uL - 5-10 mL - 1 mL - 8 mL | - <3 h - 20 min - 10 min - 80 uL/min - >30 min - > 3 h | - 30-150 nm - <200 nm - 30-200 nm - 30-100 nm - 20-600 nm - 30-200 nm | - >80% - NA - NA - >90% - 95% - 74.2% | - >97% - NA - NA - NA - 10^7^-10^8^ particles/ug protein - <20% | Han et al. 2021 (114)  Yasui et al. 2017 (107)  Wang et al. 2013 (106)  Liu et al. 2017 (105)  Woo et al. 2017 (104)  Liang et al. 2017 (103) |
| **Electrical separation** | | | | | | | |
| - Electrophoresis-driven filtration (dialysis membrane) - Electrophoresis-driven filtration (ion-selective membrane) - Ion-depletion zone microchannel - Electrophoresis-driven filtration (porous membrane) - Insulator-based dielectrophoretic - Dielectrophoretic microarray | - Plasma - Exosomes suspended in PBS, serum, & cell culture supernatant - Cell culture supernatant - Blood - Exosomes suspended in PBS - Blood | - 1 mL - 25-200 uL - NA - 240 uL - 50 uL - <25 uL | - 30 min - >10 min - 1 uL/min - 2 h - 40 min - 30 min | - 10-400 nm - 60-130 nm - NA - <500 nm - 100 nm - 20-500 nm | - 65% - 60-80% - >98% - <2% - NA - NA | - 83.6% - NA - NA - 79 ng RNA/100 ug protein - NA - NA | Cho et al. 2016 (165)  Marczak et al. 2018 (160)  Mogi et al. 2018 (159)  Davies et al. 2012 (158)  Shi et al. 2018 (156)  Lewis et al. 2018 (155) |
| **Inertial separation** | | | | | | | |
| Spiral inertial microchannel | Blood | 1000 uL | 15 min | 0.1-1 um | >52% | NA | Tay et al. 2017 (179) |
| **Viscoelastic separation** | | | | | | | |
| - Viscoelastic focusing in a reverse wavy microchannel - Viscoelastic focusing in a straight microchannel | - Cell culture supernatant - Serum & cell culture supernatant | - NA - 100 uL | - 25 uL/min - <1 h | - 30-200 nm - <200 nm | - >81% - >80% | - >92% - >90% | Zhou et al. 2019 (201)  Liu et al. 2017 (200) |
| **Deterministic lateral displacement sorting (DLD)** | | | | | | | |
| - Integrated 1024 parallel nano-DLD pillar arrays - DLD pillar arrays - Nano-DLD pillar arrays (25-235 nm pillar gap) | - Serum & urine - Cell culture supernatant - Urine | - 900 uL - 170 uL - 10 uL | - 15 uL/min - 2.5 uL/min - 0.0002 uL/min | - 30-200 nm - <250 nm - 20-110 nm | - 50% - <40% - NA | - NA - >98% - NA | Smith et al. 2018 (217)  Santana et al. 2014 (216)  Wunsch et al. 2016 (211) |
| **Field-flow fractionation (FFF)** | | | | | | | |
| - Electrical-FFF - Flow-FFF | - Exosomes suspended in PBS - Cell culture supernatant | - 46 uL - 20 uL | - <1 h - <10 min | - 120 nm - 30-100 nm | - 70-80% - NA | - NA - NA | Petersen et al. 2018 (263)  Zhang et al. 2019 (232) |
| **Pinched flow fractionation (PFF)** | | | | | | | |
| PFF in a microchannel with a pinched segment (20 um) | Cell culture supernatant | NA | 200 uL/min | 30-100 nm | NA | NA | Shin et al. 2017 (272) |
| **Acoustic separation** | | | | | | | |
| - Surface acoustic waves (20 MHz) - Sequential surface acoustic wave (20 MHz) - Acoustic trapping capillary - Surface acoustic waves (38.5 MHz) | - Plasma - Blood - Blood, urine, & cell culture supernatant - Cell culture supernatant & blood | - NA - 100 uL - 0.3-5 mL - 10-50 uL | - 0.5 uL/min - 25 min - 30 min - NA | - 20-600 nm - <150 nm - 30-500 nm - <200 nm | - NA - 82.4% - 5.7-9.3% - >80% | - NA - NA - 98.4% - NA | Wu et al. 2019 (284)  Wu et al. 2017 (283)  Evander et al. 2015 (282)  Lee et al. 2015 (281) |
| **Centrifugal separation** | | | | | | | |
| Microfluidic centrifugation at 1300 *g* | Cell culture supernatant | 5 uL | 8 min | 50-400 nm | 90% | 85% | Yeo et al. 2018 (308) |

**References**

(101) Li, P.; Kaslan, M.; Lee, S. H.; Yao, J.; Gao, Z. Progress in Exosome Isolation Techniques. Theranostics 2017, 7, 789−804.

(102) Díaz-Reinoso, B. Concentration and Purification of Seaweed Extracts Using Membrane Technologies. Sustainable Seaweed Technologies 2020, 2020, 371−390.

(103) Liang, L.-G.; Kong, M.-Q.; Zhou, S.; Sheng, Y.-F.; Wang, P.; Yu, T.; Inci, F.; Kuo, W. P.; Li, L.-J.; Demirci, U.; Wang, S. An Integrated Double-Filtration Microfluidic Device for Isolation, Enrichment and Quantification of Urinary Extracellular Vesicles for Detection of Bladder Cancer. Sci. Rep. 2017, 7, 46224.

(104) Woo, H. K.; Sunkara, V.; Park, J.; Kim, T. H.; Han, J. R.; Kim, C. J.; Choi, H. Il; Kim, Y. K.; Cho, Y. K. Exodisc for Rapid, Size- Selective, and Efficient Isolation and Analysis of Nanoscale Extracellular Vesicles from Biological Samples. ACS Nano 2017, 11, 1360−1370.

(105) Liu, F.; Vermesh, O.; Mani, V.; Ge, T. J.; Madsen, S. J.; Sabour, A.; Hsu, E. C.; Gowrishankar, G.; Kanada, M.; Jokerst, J. V.; Sierra, R. G.; Chang, E.; Lau, K.; Sridhar, K.; Bermudez, A.; Pitteri, S. J.; Stoyanova, T.; Sinclair, R.; Nair, V. S.; Gambhir, S. S.; Demirci, U. The Exosome Total Isolation Chip. ACS Nano 2017, 11, 10712−10723.

(106) Wang, Z.; Wu, H. J.; Fine, D.; Schmulen, J.; Hu, Y.; Godin, B.; Zhang, J. X. J.; Liu, X. Ciliated Micropillars for the Microfluidic-Based Isolation of Nanoscale Lipid Vesicles. Lab Chip 2013, 13, 2879−2882.

(107) Yasui, T.; Yanagida, T.; Ito, S.; Konakade, Y.; Takeshita, D.; Naganawa, T.; Nagashima, K.; Shimada, T.; Kaji, N.; Nakamura, Y.; Thiodorus, I. A.; He, Y.; Rahong, S.; Kanai, M.; Yukawa, H.; Ochiya, T.; Kawai, T.; Baba, Y. Unveiling Massive Numbers of Cancer-Related Urinary-MicroRNA Candidates via Nanowires. Science Advances 2017, 3, e1701133.

(114) Han, Z.; Peng, C.; Yi, J.; Zhang, D.; Xiang, X.; Peng, X.; Su, B.; Liu, B.; Shen, Y.; Qiao, L. Highly Efficient Exosome Purification from Human Plasma by Tangential Flow Filtration Based Microfluidic Chip. Sens. Actuators, B 2021, 333, 129563.

(155) Lewis, J. M.; Vyas, A. D.; Qiu, Y.; Messer, K. S.; White, R.; Heller, M. J. Integrated Analysis of Exosomal Protein Biomarkers on Alternating Current Electrokinetic Chips Enables Rapid Detection of Pancreatic Cancer in Patient Blood. ACS Nano 2018, 12, 3311−3320.

(156) Shi, L.; Rana, A.; Esfandiari, L. A Low Voltage Nanopipette Dielectrophoretic Device for Rapid Entrapment of Nanoparticles and Exosomes Extracted from Plasma of Healthy Donors. Sci. Rep. 2018, 8, 6751.

(158) Davies, R. T.; Kim, J.; Jang, S. C.; Choi, E.-J.; Gho, Y. S.; Park, J. Microfluidic Filtration System to Isolate Extracellular Vesicles from Blood. Lab Chip 2012, 12, 5202−5210.

(159) Mogi, K.; Hayashida, K.; Yamamoto, T. Damage-Less Handling of Exosomes Using an Ion-Depletion Zone in a Micro- channel. Anal. Sci. 2018, 34, 875−880.

(160) Marczak, S.; Richards, K.; Ramshani, Z.; Smith, E.; Senapati, S.; Hill, R.; Go, D. B.; Chang, H. C. Simultaneous Isolation and Preconcentration of Exosomes by Ion Concentration Polarization. Electrophoresis 2018, 39, 2029−2038.

(165) Cho, S.; Jo, W.; Heo, Y.; Kang, J. Y.; Kwak, R.; Park, J. Isolation of Extracellular Vesicle from Blood Plasma Using Electrophoretic Migration through Porous Membrane. Sens. Actuators, B 2016, 233, 289−297.

(179) Tay, H. M.; Kharel, S.; Dalan, R.; Chen, Z. J.; Tan, K. K.; Boehm, B. O.; Loo, S. C. J.; Hou, H. W. Rapid Purification of Sub- Micrometer Particles for Enhanced Drug Release and Microvesicles Isolation. NPG Asia Mater. 2017, 9, e434.

(200) Liu, C.; Guo, J.; Tian, F.; Yang, N.; Yan, F.; Ding, Y.; Wei, J.; Hu, G.; Nie, G.; Sun, J. Field-Free Isolation of Exosomes from Extracellular Vesicles by Microfluidic Viscoelastic Flows. ACS Nano 2017, 11, 6968−6976.

(201) Zhou, Y.; Ma, Z.; Tayebi, M.; Ai, Y. Submicron Particle Focusing and Exosome Sorting by Wavy Microchannel Structures within Viscoelastic Fluids. Anal. Chem. 2019, 91, 4577−4584.

(211) Wunsch, B. H.; Smith, J. T.; Gifford, S. M.; Wang, C.; Brink, M.; Bruce, R. L.; Austin, R. H.; Stolovitzky, G.; Astier, Y. Nanoscale Lateral Displacement Arrays for the Separation of Exosomes and Colloids Down to 20 Nm. Nat. Nanotechnol. 2016, 11, 936−940.

(216) Santana, S. M.; Antonyak, M. A.; Cerione, R. A.; Kirby, B. J. Microfluidic Isolation of Cancer-Cell-Derived Microvesicles from Hetergeneous Extracellular Shed Vesicle Populations. Biomed. Micro- devices 2014, 16, 869−877.

(217) Smith, J. T.; Wunsch, B. H.; Dogra, N.; Ahsen, M. E.; Lee, K.; Yadav, K. K.; Weil, R.; Pereira, M. A.; Patel, J. V.; Duch, E. A.; Papalia, J. M.; Lofaro, M. F.; Gupta, M.; Tewari, A. K.; Cordon-Cardo, C.; Stolovitzky, G.; Gifford, S. M. Integrated Nanoscale Deterministic Lateral Displacement Arrays for Separation of Extracellular Vesicles from Clinically-Relevant Volumes of Biological Samples. Lab Chip 2018, 18, 3913−3925.

(232) Zhang, H.; Lyden, D. Asymmetric-Flow Field-Flow Fractionation Technology for Exomere and Small Extracellular Vesicle Separation and Characterization. Nat. Protoc. 2019, 14, 1027−1053.

(263) Petersen, K. E.; Shiri, F.; White, T.; Bardi, G. T.; Sant, H.; Gale, B. K.; Hood, J. L. Exosome Isolation: Cyclical Electrical Field Flow Fractionation in Low-Ionic-Strength Fluids. Anal. Chem. 2018, 90, 12783−12790.

(272) Shin, S.; Han, D.; Park, M. C.; Mun, J. Y.; Choi, J.; Chun, H.; Kim, S.; Hong, J. W. Separation of Extracellular Nanovesicles and Apoptotic Bodies from Cancer Cell Culture Broth Using Tunable Microfluidic Systems. Sci. Rep. 2017, 7, 9907.

(281) Lee, K.; Shao, H.; Weissleder, R.; Lee, H. Acoustic Purification of Extracellular Microvesicles. ACS Nano 2015, 9, 2321−2327.

(282) Evander, M.; Gidlöf, O.; Olde, B.; Erlinge, D.; Laurell, T. Non-Contact Acoustic Capture of Microparticles from Small Plasma Volumes. Lab Chip 2015, 15, 2588−2596.

(283) Wu, M.; Ouyang, Y.; Wang, Z.; Zhang, R.; Huang, P.-H.; Chen, C.; Li, H.; Li, P.; Quinn, D.; Dao, M.; Suresh, S.; Sadovsky, Y.; Huang, T. J. Isolation of Exosomes from Whole Blood by Integrating Acoustics and Microfluidics. Proc. Natl. Acad. Sci. U. S. A. 2017, 114, 10584−10589.

(284) Wu, M.; Chen, C.; Wang, Z.; Bachman, H.; Ouyang, Y.; Huang, P. H.; Sadovsky, Y.; Huang, T. J. Separating Extracellular Vesicles and Lipoproteins via Acoustofluidics. Lab Chip 2019, 19, 1174−1182.

(308) Yeo, J. C.; Kenry; Zhao, Z.; Zhang, P.; Wang, Z.; Lim, C. T. Label-Free Extraction of Extracellular Vesicles Using Centrifugal Microfluidics. Biomicrofluidics 2018, 12, 024103.

ß
